# Immunoinformatic-driven design and evaluation of multi-epitope mRNA vaccine targeting HIV-1 gp120

**DOI:** 10.3389/fimmu.2025.1480025

**Published:** 2025-05-13

**Authors:** Muhammad Zeeshan Ahmed, Tazeen Rao, Zeeshan Mutahir, Sarfraz Ahmed, Najeeb Ullah, Suvash Chandra Ojha

**Affiliations:** ^1^ Department of Biochemistry, Bahauddin Zakariya University, Multan, Pakistan; ^2^ School of Biochemistry and Biotechnology, University of the Punjab, Lahore, Pakistan; ^3^ Wellman Center for Photomedicine, Massachusetts General Hospital, Harvard Medical School, Boston, MA, United States; ^4^ Department of Infectious Diseases, The Affiliated Hospital of Southwest Medical University, Luzhou, Sichuan, China

**Keywords:** aids, epitopes, gp120, HIV-1 infection, immunoinformatics, mRNA-vaccine peptides

## Abstract

HIV (human immunodeficiency virus) presents a global health crisis, causing significant AIDS-related deaths and over one million new infections annually. The curbing of HIV is an intricate and continuously evolving domain, marked by numerous challenges, including drug resistance and the absence of a significant cure or vaccine because of its mutating ability and diverse antigens in its envelope, prompting research for functional cures and long-term remission strategies. The endeavor to devise an HIV vaccine capable of eliciting robust and broadly cross-reactive humoral and cellular immune responses is a formidable undertaking, primarily due to the pronounced genetic heterogeneity of HIV-1, the variances observed in virus subtypes (clades) across distinct geographic regions, and the polymorphic nature of human leukocyte antigens (HLA). The viral envelope protein (gp120) selectively interacts with CD4 and chemokine receptors on the surface of target cells. It serves as the key initiator in the intricate viral entry into host cells, rendering it a compelling candidate for vaccine development. This study used bioinformatic tools to design a safe, hypoallergenic, and non-toxic mRNA HIV-1 vaccine by assembling immunogenic B- and T-cell epitopes from the gp120 protein. We identified antigenic, non-toxic, and non-allergic B-cell epitopes (IEPLGIAPTRAKRRVVER) and T-cell epitopes (QQKVHALFY, ITIGPGQVF, WQGVGQAMY, APTRAKRRV, KQQKVHALFYRLDIV, QQKVHALFYRLDIVQ, QKVHALFYRLDIVQI, SLAEEEIIIRSENLT, and IRSENLTNNVKTIIV). For designing the mRNA vaccine against HIV-1 gp120, we assembled the epitopes with 5′ m7G cap, 5′ UTR (untranslated region), Kozak sequence, signal peptide (tPA), RpfE (resuscitation-promoting factor E) adjuvant at N-terminal and MITD (MHC class I trafficking domain) adjuvant, stop codon, 3′ UTR, and 120-nucleotide long poly(A) tail at the C-terminal with immunogenic robustness linkers. The mRNA vaccine is translated into a protein-based vaccine by the host body’s ribosomes. Their comprehensive computational findings, including physicochemical, structural, and 3D refinement analyses, substantiated the stability and quality of the translated vaccine. Molecular docking and simulation revealed a strong and stable binding affinity of vaccine immunization with immune cells’ pattern recognition receptors (TLR4). Immune simulations demonstrated a potent primary immune response characterized by a gradual increase in immunoglobulins and a corresponding decline in antigen concentration. This bioinformatics-driven study presents a promising HIV-1 mRNA vaccine candidate, underscoring the need for further experimental validation through preclinical and clinical trials. At the same time, its methodologies hold the potential for addressing other challenging infectious diseases, thereby impacting vaccinology broadly.

## Graphical Abstract

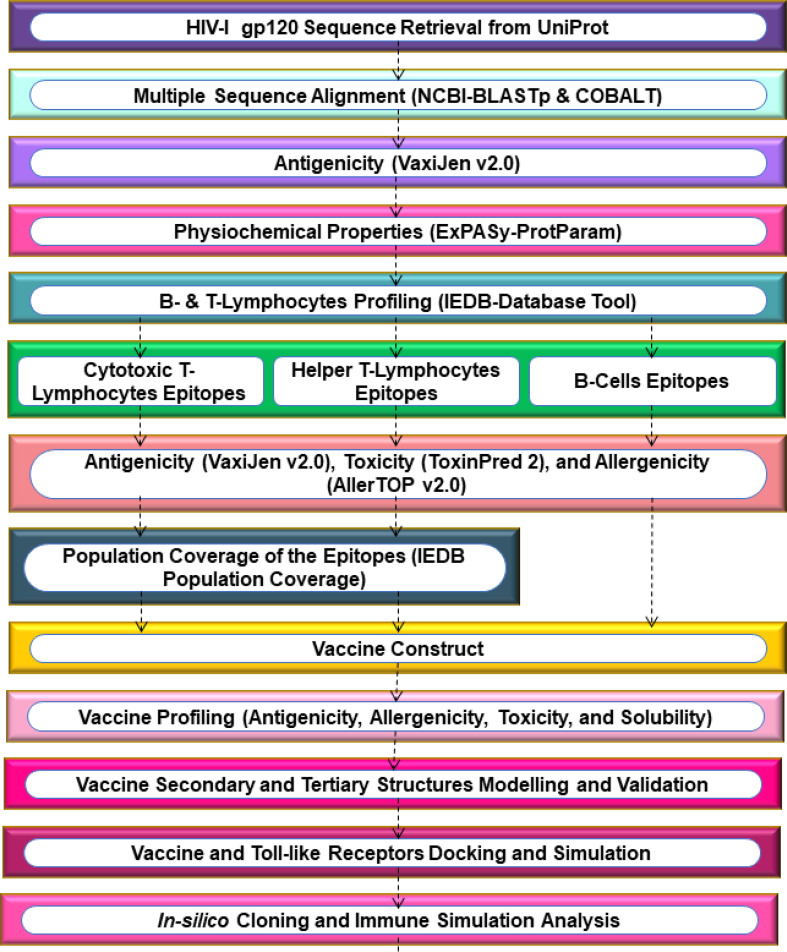

## 1 Introduction

Human Immunodeficiency Virus (HIV) is a pathogen that exerts a debilitating effect on the host’s immune system, rendering it susceptible to a spectrum of infections and ailments, culminating in the development of acquired immunodeficiency syndrome (AIDS), which represents the most advanced stage of HIV infection. Recent statistics reported by the World Health Organization (WHO) indicate about 38.4 million individuals globally in 2021 were grappling with HIV infection (1, 2). This viral infection also accounted for the demise of around 650,000 individuals in 2021 due to HIV-related complications (3). A definitive cure for HIV infection remains elusive, and therapeutic intervention in the form of antiretroviral therapy (ART) has substantially mitigated the impact of the virus. HIV exerts its deleterious effects by selectively targeting CD4+ T lymphocytes (CD4 T cells), a subset of immune cells vital for orchestrating defense mechanisms against infections and diseases (4). The destruction of CD4 T cells by HIV results in a compromised immune system, rendering the individual highly susceptible to opportunistic infections, which encompass disorders such as bacterial infections, fungal infections, tuberculosis, and malignancies. Furthermore, HIV inflicts neurological damage, leading to a spectrum of neurological impairments, including pain, numbness, muscle weakness, and mobility challenges (5). Antiretroviral drugs constitute the cornerstone of HIV management. These pharmacological agents are instrumental in suppressing viral replication, thereby reducing the viral load in the bloodstream and forestalling further immune system deterioration. Despite these therapeutic strides, HIV infection endures in latent reservoirs within specific white blood cells, such as myeloid cells, monocytes, and macrophages, even when the virus is undetectable in the bloodstream. This aspect underscores the chronicity of HIV infection and the necessity for continued therapeutic vigilance (6).

ART involves taking a specific combination of medications every day to fight and manage the HIV infection. This therapy serves the dual purpose of curtailing the viral load within the host’s body and sustaining overall health (4). The pharmacological arsenal against HIV comprises various classes of drugs, each with distinct mechanisms of action. These encompass reverse transcriptase inhibitors, integrase inhibitors, protease inhibitors, and entry or fusion inhibitors, collectively targeting discrete phases of the HIV life cycle, thereby impeding its replication and propagation (7). ART is universally recommended for all individuals diagnosed with HIV and should be initiated expeditiously following diagnosis to optimize its therapeutic benefits (7). Nevertheless, anti-HIV drugs do not confer a curative solution for HIV infection. Consequently, their administration requires strict adherence and should follow healthcare provider-prescribed regimens. WHO is actively engaged in optimizing antiretroviral therapies to enhance efficacy, safety, and tolerability across diverse populations (8). While crucial in handling HIV, ART is not without potential side effects. These may encompass adverse reactions such as nausea, fatigue, headaches, and skin rashes (9). The importance of ongoing medical monitoring and management when implementing ART as part of HIV treatment protocols.

Vaccination is a crucial method for preventing infectious diseases. It works by prompting the immune system to produce antibodies (Abs) that can fight off harmful invaders (10). HIV vaccines are categorized into two hold relevance: preventive vaccines and therapeutic vaccines. Prophylactic vaccines are designed to safeguard individuals without HIV infection from becoming infected (11). Conversely, therapeutic vaccines aim to fortify the immune responses of individuals already living with HIV, assisting them in controlling the virus (12, 13). Despite concerted research efforts (14–16), the development of an effective HIV vaccine remains an unmet challenge. The formidable obstacle arises from the rapid mutability of HIV, especially in its surface proteins’ capacity to elude immune detection, necessitating the design of a vaccine capable of targeting diverse HIV strains and eliciting robust and enduring immune responses (17, 18). Consequently, individuals living with HIV are encouraged to undergo vaccinations against other disorders to which they may be more susceptible, including but not limited to COVID-19, HPV, influenza, hepatitis B, and polio, to avert complications and enhance their overall health outcomes (19, 20).

Hence, HIV envelope proteins exhibit high mutability. The glycoprotein (gp)120, situated on the virus envelope, plays a pivotal role in mediating viral attachment and entry into the host cell via the CD4 receptor, as well as immune evasion mechanisms. Compounds designed to target gp120, referred to as attachment inhibitors, function by obstructing the interaction between HIV and CD4 T cells, thereby preventing viral entry (21, 22). Notably, the sole approved drug within this class is Fostemsavir (Rukobia), a prodrug of Temsavir reserved for individuals grappling with multidrug-resistant HIV and limited therapeutic alternatives. Fostemsavir administration may be associated with side effects, as well as potential drug interactions necessitating consultation with healthcare providers before its use (23). The development of effective HIV vaccines faces significant challenges, as trials like RV144 have shown modest efficacy (31%), while others, such as the Mosaico study, have failed to offer substantial protection despite being safe for participants (24, 25). The extraordinary genetic diversity of HIV, with multiple clades and a high mutation rate, complicates efforts to create a universal vaccine capable of cross-reacting with various viral strains (26, 27). Current vaccines struggle to elicit broadly neutralizing Abs (bnAbs) or robust immune responses, as immune correlates of protection remain unclear (24, 26, 28). Some candidates have even increased the risk of infection, as seen in the Merck Ad5 trial, where vaccinated individuals exhibited higher rates of infection. Additionally, HIV’s rapid establishment of latent reservoirs evades immune detection and antiretroviral therapy, further complicating vaccine strategies. A decline in research funding and interest compared to other diseases, such as COVID-19, exacerbates these issues, potentially hindering the development of innovative solutions. Despite progress, significant weaknesses in efficacy, immune response, and addressing viral diversity and latency hinder successful vaccine development (29).

Vaccines targeting HIV gp120 induce bnAbs obstructing gp120’s interaction with the CD4 receptor, thus thwarting HIV infection (30). The primary challenge stems from the pronounced variability of gp120, which allows it to evade immune recognition. As a result, researchers are exploring diverse strategies to enhance the immunogenicity and comprehensiveness of gp120-based vaccines, including the utilization of trimeric gp120 forms, amalgamation with other antigens or adjuvants, or the targeting of alternative regions within the HIV envelope (31). Recent advances in HIV vaccine development have demonstrated both progress and persistent challenges in gp120-based strategies. Clinical trials evaluating different formulations have highlighted incremental improvements in immunogenicity while emphasizing the need for broader immune responses. HVTN 107 assessed ALVAC-HIV prime-boost regimens with gp120 adjuvanted by MF59 or alum, revealing that both adjuvants elicited 100% gp120-binding IgG responses without significant differences in magnitude or breadth, suggesting that adjuvant selection may be less critical than previously assumed (32). Similarly, HVTN 108 compared a DNA prime (DNA-HIV-PT123) with AS01B-adjuvanted gp120, demonstrating superior Env gp120 IgG response rates, enhanced V1V2 IgG magnitude, and increased Fc-mediated functions relative to MF59, positioning AS01B as a promising adjuvant for V1V2-directed immunity (33). These align with RV144 follow-ups, which linked V1V2-specific antibodies to reduced HIV risk, prompting the development of multivalent gp120 vaccines such as the pentavalent subtype C formulation—designed using computationally selected Envs and adjuvanted with alum or MF59, achieving broader V1V2 IgG coverage (34, 35).

Earlier trials, such as VAX004, underscored the limitations of bivalent subtype B gp120 against circulating strains, shaping today’s computationally guided, multivalent approaches. Preclinical innovations further support these refinements; for instance, sugar-shield targeting involved gp120 fragments conjugated to conserved sugar molecules, inducing cross-reactive antibodies but failing to achieve robust neutralization (36), while gp120-CD4 chimeras, such as IHV01, demonstrated the feasibility of eliciting broadly reactive antibodies against conserved CD4-induced epitopes (37). The pentavalent subtype C vaccine, incorporating CAP260/CAP174/Ko224 alongside TV1c8/1086C gp120, significantly expanded V1V2 IgG breadth (from 83% coverage of heterologous strains to 67% in trivalent formulations) and improved linear epitope recognition at conserved V2.2 sites (35). Another study evaluated whether an ALVAC prime with computationally selected gp120 boosts, aimed at maximizing subtype C diversity, could enhance protection in non-human primates. The study found that a trivalent subtype C gp120 boost significantly protected NHPs from repeated SHIV challenges, with antibody-dependent cellular cytotoxicity identified as a key correlate of protection. These findings suggest that computationally optimized vaccines focusing on V1V2 coverage could improve HIV vaccine efficacy (38). Despite these advancements, challenges remain: adjuvant trade-offs persist, as MF59 enhances immunogenicity but exhibits only marginal advantages over cost-effective alum in trials like HVTN 107 (32, 34); multivalent formulations still struggle to neutralize diverse HIV strains, underscoring the necessity for epitope-focused designs (35, 39); and durability beyond 12 months remains unproven in newer regimens.

The limited ability of gp120-based HIV vaccines to elicit bnAbs arises from persistent structural and immunological challenges. Monomeric gp120 lacks the native trimeric conformation of the viral envelope spike, preventing the presentation of critical quaternary epitopes targeted by bnAbs such as VRC01 and PG9 (40, 41). Although SOSIP-stabilized trimers more effectively expose conserved epitopes like the CD4-binding site and V2 apex, they still struggle to induce cross-clade neutralization (42). Furthermore, the glycan shield and hypervariable loops (V1/V2, V3) dominate the immune response, diverting antibodies toward non-neutralizing or strain-specific targets (40, 43). Engineered gp120 mutants, such as mCHO variants with additional glycans, have been developed to suppress immunodominant non-neutralizing epitopes, but they fail to enhance bnAb responses (44). Additionally, immune complexes formed by gp120 and anti-CD4bs monoclonal antibodies improve V3-directed neutralization in murine models, yet these responses remain narrow and lack cross-clade breadth (43). Even with adjuvants such as MF59 or AS01B, gp120-based immunogens primarily stimulate short-lived plasma cells, limiting bnAb maturation (41, 45).

Current strategies to overcome these limitations include native-like trimers (e.g., BG505 SOSIP) that mimic the viral spike but elicit only autologous tier 2 neutralization. Germline-targeting immunogens, such as eOD-GT8, show promise in priming VRC01-class B cells in early clinical trials; however, effective affinity maturation remains a challenge. Glycan engineering approaches, such as selective removal of glycosylation sites (e.g., N332), aim to expose conserved high-mannose patches, but these modifications risk destabilizing the Env trimer (Haynes et al.). The immune complexes to focus Abs responses have demonstrated enhanced V3-directed neutralization but have failed to induce CD4bs-specific bnAbs (43). Lessons from gp120-based vaccine trials have informed modern immunogen design strategies, including epitope focusing via stabilized transitional Env states (46)glycan hole engineering to expose conserved neutralization sites (Haynes et al.; 43), and population-specific vaccine tailoring, given demographic variability in immune responses (45). While gp120 alone is insufficient for bnAb induction, its structural insights contribute to hybrid vaccination approaches, such as prime-boost regimens utilizing germline-targeting primers followed by SOSIP trimer boosts, chimeric immunogens combining gp120-CD4 complexes with stabilized trimers, and computationally designed mosaic nanoparticles incorporating gp120-derived conserved epitopes (46).

Traditional vaccine development faces significant challenges, including the complexity of antigen selection for diverse and evasive viruses like HIV, lengthy and costly development processes, and logistical hurdles in production, storage, and distribution, particularly in resource-limited settings and public health emergencies (26, 47, 48). In contrast, mRNA vaccines offer several advantages, including rapid development, versatility in targeting different pathogens, reduced production time, and increased safety compared to DNA vaccines, killed vaccines, and live attenuated vaccines (49). More importantly, artificial intelligence (AI) facilitates vaccine design by predicting epitopes, identifying genomic regions, and accelerating the research and development of vaccines. By leveraging AI, researchers can expedite the identification of potential vaccines, optimize vaccine design, and contribute to the rapid creation of effective vaccines, particularly for emerging biological threats (50–53).

*In silico* vaccine design for HIV, gp120 entails the utilization of computational tools and models to conceptualize or select prospective vaccine candidates that specifically target the gp120 protein of HIV (54–57). A noteworthy example of this approach involves the *in silico* selection of ssDNA aptamers with the capacity to bind to gp120, inhibiting HIV-1 infection. Aptamers, characterized by their ability to adopt distinct structural configurations and recognize diverse molecular targets, undergo a selection process that leverages bioinformatics algorithms to generate and sift through numerous aptamer sequences. Such selection strategies, guided by affinity, specificity, stability, and functionality criteria, offer potential advantages in terms of resource and time efficiency when contrasted with traditional SELEX methodologies, which necessitate multiple rounds of *in vitro* selection and amplification (58). Additionally, *in silico*-designed HIV-1 vaccine candidates have been validated through *in vivo* studies, demonstrating their ability to elicit strong immune responses. Notably, multiepitope-based DNA and polypeptide vaccines targeting conserved HIV-1 regions induced significant IFN-γ and Granzyme B production in mice, supporting the feasibility of computationally designed vaccines for real-world application. (59–61).

Our study presents a novel immunoinformatics-driven approach to developing a multi-epitope mRNA vaccine targeting the HIV-1 gp120 protein, which integrates multiple antigenic elements to enhance the immune response. The research detailed herein aims to develop a hypoallergenic, non-toxic, and innocuous vaccine against HIV-1 using artificial intelligence. This vaccine combines multiple antigenic linear epitopes characterized by their non-allergenic properties, immunogenic potential, and ability to induce cytokine responses. We prioritize linear epitopes for their computational predictability and conservation across HIV strains, making them ideal for universal vaccine design. Despite the dominance of conformational epitopes in natural immunity, linear epitopes enhance B-cell priming, facilitate affinity maturation, and improve T-cell collaboration for a stronger immune response. These constituents, derived from the gp120 protein of HIV-1, are subject to comprehensive analysis using bioinformatic tools, including molecular docking, simulations, and immune simulations, to activate the immune system and evaluate their real-world immunogenic performance. The vaccine candidates were selected for their hypoallergenic and nontoxic profiles, ensuring patient safety while minimizing adverse reactions. Advanced bioinformatics tools were utilized to analyze epitope interactions with immune receptors, thereby enhancing the vaccine’s capacity to stimulate both humoral and cellular immunity. By incorporating adjuvants and specific linkers, the design promotes robust activation of both T cells and B cells, offering potential for long-term protection. Real-world simulations were conducted to assess clinical applicability, bridging the gap between theoretical designs and practical viability. Furthermore, this methodology provides a foundation for designing vaccines against other infectious diseases by leveraging conserved epitopes and optimizing immune responses.

## 2 Methodology

### 2.1 Protein sequence retrieval and multiple sequence alignment

The HIV-1 gp120 sequence was retrieved from the UniProt database (UniProt ID: Q9IZE4), as reported in a clinical trial (62). This sequence represents a well-characterized HIV-1 strain with structural and functional relevance to vaccine studies (63). The gp120 sequence was searched on BLASTp to obtain a highly similar sequence. The ≥80% of identical gp120 sequences were aligned using COBALT (Constraint-based Multiple Alignment Tool) to identify conserved regions in the HIV-1 gp120 sequence. This process leveraged both local sequence similarity and conserved domain information.

### 2.2 Antigenicity and physicochemical properties of the gp120 protein

The antigenicity of the conserved HIV-1 gp120 sequence was assessed using the VaxiJen v2.0 server with a default threshold. VaxiJen’s default threshold is 0.5, considering regions with scores ≥0.5 as antigenic, based on the model’s strong performance in achieving high accuracy (70% to 89%) and balanced precision and recall on a validation dataset. The stability and reliability of VaxiJen make it a valuable tool for antigen prediction in reverse vaccinology (64–66). After confirming the antigenicity, the physicochemical properties of the HIV-1 gp120 sequence were assessed using the ExPASy-ProtParam online server (67, 68). ProtParam, available within this platform, computes various physical and chemical parameters for protein sequence, either user-provided or retrieved from TrEMBL and Swiss-Prot databases. These parameters encompass the count of amino acids, molecular weight (MW) in kilodaltons, theoretical pI (isoelectric point), extinction coefficient (Ec) measured in M-1 cm-1 at 280 nm, and estimated half-life.

### 2.3 Epitopes prediction and important features profiling

The T-cell and B-cell epitopes were predicted using the IEDB (Immune Epitope Database) (69). IEDB employs T-cell epitope prediction, antibody (Ab) initio prediction, B-cell epitope prediction, homology-based prediction, and structure-based prediction to curate, forecast, and validate epitopes based on reported literature and experimental data. This study utilized the MHC-I binding prediction server with the NetMHCpan EL 4.1 method (70) to predict MHC-I binding epitopes in the HIV-1 gp120 sequence. The FASTA sequence of gp120 was submitted to the MHC-I binding prediction server, with MHC species *Homo sapiens* and 9-mer HLA allele reference set.

Similarly, using the IEDB-recommended 2.22 method (69)The MHC-II binding prediction server was utilized to predict MHC-II binding epitopes within the HIV-1 gp120 sequence. The FASTA sequence of gp120 was separately submitted to the MHC-II binding prediction server, using H. sapiens as the species for loci HLA-DR, HLA-DQ, and HLA-DP, with a complete reference HLA set and a default 15-mer epitope length. Furthermore, using the BepiPred Linear Epitope Prediction 2.0 method (71)The Ab epitope prediction server was employed to predict B-cell epitopes for vaccine construction in the HIV-1 gp120 sequence.

Subsequently, all epitopes predicted by epitope prediction servers were organized in an Excel spreadsheet. Each predicted epitope was evaluated to ensure its inclusion in the conserved sequence and its presence outside the transmembrane regions of the HIV-1 gp120 sequence. These epitopes were then profiled to predict their antigenicity, toxicity, and allergenicity using the VaxiJen v2.0 (64–66), ToxinPred2 (72), and AllerTOP v2.0 (73) Servers, respectively. Population coverage was calculated to estimate the percentage of the South Asian population, specifically from India and Pakistan, predicted to be covered by individuals with MHC class I and II epitopes. These calculations relied on IEDB’s Population Coverage tool (74). Further, a comprehensive conservancy was analyzed on all selected antigenic epitopes derived from the HIV-1 gp120 sequence (75).

### 2.4 Vaccine construction, characterization, and structures prediction

The vaccine sequence was initiated with an adjuvant, RpfE (resuscitation-promoting factor E), at the N-terminus, connected by an EAAAK linker. Subsequently, all the recruited antigenic, non-allergenic, and non-toxic B-cells, MHC-II, and MHC-I epitopes were assembled with AAY, GPGPG, and KK linkers, respectively (76–78). Lastly, the vaccine construct was concluded with the MITD (MHC class I trafficking domain) sequence (UniProt ID: Q8WV92) (79–81), and a 6-His tag (82) At the C-terminal.

For the development of an mRNA vaccine against HIV-1, we reverse-translated the above sequence of protein-based vaccine to an optimized sequence of RNA, adhering to established protocols (Mohammadi et al.; 81, 83, 84). The online server for the Java Codon Adaptation Tool (JCAT) enabled the optimization of codons to improve vaccine expression. This included considerations such as effective ribosome binding, sites for restriction enzyme cleavage, and transcription termination, (85). Additionally, the mRNA vaccine construct included features such as a 5′ m7G cap, 5′ UTR (untranslated region), Kozak sequence, and signal peptide (tPA) (UniProt ID: P00750) at the N-terminal, and stop codon, 3′ UTR, and 120-nucleotide long poly(A) tail at the C-terminal. The RNAfold web server then predicted the mRNA secondary structure and thermodynamically determined the minimal free energy score (86–88).

The mRNA vaccine is capable of being translated into antigenic proteins within immune cells, thereby activating adaptive immune responses. Additionally, the vaccine triggers innate immunity, stimulating the production of antibodies (Abs) and inducing long-term cellular immunity. This presents a promising alternative to conventional vaccines (89, 90). The physicochemical characteristics of the translated protein vaccine were assessed using the ExPASy-ProtParam online server (67, 68). The allergenicity, antigenicity, toxicity, and solubility of the vaccine were estimated using AllerTop 2.0 (73), ANTIGENpro (91, 92), VaxiJen 2.0 (64–66), Toxinpred2 (72), and SoluProt tool (93), respectively. The solubility scores of vaccine expression in *Escherichia coli* exceeding 0.5 indicate a soluble protein, while those below 0.5 suggest an insoluble protein.

Various parameters related to the secondary structure of the HIV-1 vaccine were determined by SOPMA (94). Default settings were applied for SOPMA, including the use of four conformational states (helix, sheet, turn, and coil), an 8.0 similarity threshold, and a 17-window width. For a more detailed graphical representation of the vaccine’s secondary structure, the PSIPRED (PSI-blast-based secondary structure prediction tool) (95–97) was employed to analyze the combined vaccine construct of HIV-1. Subsequently, the tertiary structure of the translated vaccine was built using ColabFold (98), which utilizes AlphaFold2 and Alphafold2-multimer to generate sequence templates through HHsearch and MMseqs2 methods.

### 2.5 Refinement and verification of the 3D structure of the vaccine

Hence, the 3D-predicted structure of the HIV-1 gp120 vaccine underwent refinement using the GalaxyRefine online web server (99). This server conducted repacking and side-chain rebuilding processes to relax the structure through molecular dynamic simulations (MDS). To assess the quality of the 3D structure of the vaccine construct, validation was performed using PROCHECK (100). PROCHECK assesses the stereochemical quality of a protein structure by analyzing the geometry and overall structure of each residue, generating a Ramachandran Plot.

### 2.6 Discontinuous B-cell epitope prediction

The existence of conformational and linear B-cell epitopes within the constructed vaccine was verified using the ElliPro tool, which provides PI (Protrusion Index) scores for each epitope (101). The scores are derived from the geometric characteristics of the protein structure, utilizing calculations involving the distances and angles between atoms within the protein structure. A higher score indicates a greater likelihood that the specific region is an epitope (102, 103).

### 2.7 Molecular docking and molecular dynamic simulation

To assess the binding affinity between the vaccine and immune cell receptor, i.e., Toll-like receptor 4 (TLR4), a protein-protein molecular docking analysis was conducted using ClusPro 2.0 (104–106). The TLR4 is significant in its essential role in initiating adaptive immune responses by the vaccine, boosting the vaccine’s overall efficacy (107, 108). The TLR4 3D crystal structure was retrieved from the Protein Data Bank (PDB) (PDB ID: 4G8A). Before docking, all ligands and other heteroatoms were removed from the TLR4 structure. Subsequently, the purified TLR4 protein was designated as the receptor, while the vaccine was used as a ligand, and both were submitted to the ClusPro 2.0 server for protein-protein docking (109, 110).

The most favorable vaccine-TLR4 docked, analyzed the normal mode analysis (NMA) through the iMODS online server (111). The iMODS server uses NMA to study the coordinated movement of internal coordinates and torsional angles in the vaccine-TLR4 complex. Essential dynamics help assess protein stability and predict the complex’s natural movements, considering factors like B-factors, covariance, deformability, and eigenvalues. Essential dynamics are a cost-effective alternative to atomistic simulations, aiding in the evaluation of protein stability and the prediction of the complex’s inherent motions.

The Desmond software application, developed by Schrödinger LLC in New York, United States, performed a 100-nanosecond (ns) molecular dynamics simulation (MDS) of the HIV-1 gp120 vaccine-TLR4 complex. During the MDS, Newton’s classical equation of motion was applied to predict the status of protein-protein binding under physiological conditions (112–117). During the MDS experiments, we optimized and minimized the interactions between the vaccine and TLR4 using Maestro’s Protein Preparation Wizard to ensure that there were no steric conflicts, poor contacts, or distorted geometries. The systems were built using the System Builder tool, and we utilized the TIP3P (Intermolecular Interaction Potential 3 Points Transferable) as the solvent model in an orthorhombic box with the OPLS_2005 force field (118, 119). To replicate physiological environments, simulation conditions were set at 300 K and 1 atm pressure. Counterions were added to neutralize the docked complex model, and a 0.15 M sodium chloride solution was prepared. Trajectories were captured at 100 picoseconds (ps) intervals. The stability of the vaccine-TLR4 interactions was assessed by monitoring the Root Mean Square Deviation (RMSD), Root Mean Square Fluctuation (RMSF), hydrogen contacts, and changes in secondary structure elements (SSE) over time (112–117).

### 2.8 Molecular mechanics and generalized born surface area calculations

The binding free energy (ΔG_bind_) of the vaccine-TLR4 complex during the MDS was calculated using the Prime module of the MM-GBSA (molecular mechanics generalized Born surface area) approach. This calculation employed rotamer search techniques, the OPLS 2005 force field, and the VSGB solvent model. MD trajectory frames were selected every 10 ns of the simulation run. The overall ΔG_bind_ was then determined using the following Equation 1:


(1) ΔGbind=Gcomplex−(Greceptor+Gvaccine)

where G_bind_ = binding-free energy, G_complex_ = docked complex-free energy, G_vaccine_ = vaccine-free energy, and G_receptor_ = TLR4 receptor-free energy.

The ΔG_bind_ was calculated using Equation 2, which includes contributions from the molecular mechanics of gas-phase energy (ΔEgas), solvation-free energy (ΔG_sol_), and entropy (TΔS).


(2) ΔGbind=ΔEɡas+ΔGsol−TΔS

Where ΔE_gas_ = van der Waals and electrostatic interactions, excluding internal energy. ΔG_sol_ = nonpolar (solvent-accessible surface area) + polar (generalized Born) components. TΔS = conformational entropy, including rotational, translational, and vibrational entropy changes upon binding.

### 2.9 Codon optimization and *in-silico* cloning

To facilitate the efficient expression of the vaccine in *E. coli* K12, we employed JCAT (85). This tool optimized codon usage, ensuring the vaccine sequence’s compatibility with the prokaryotic ribosome’s binding site, avoiding restriction enzyme cleavage sites, and ensuring rho-independent transcription termination signals. Following codon adaptation, SnapGene 4.2 software (110, 120)was employed to facilitate the cloning process. To facilitate cloning, PshAI and BgLI restriction sites, which are present in both the vector and the vaccine sequence, were introduced at the N- and C-terminals of the vaccine sequence, respectively. The resulting vaccine construct was inserted into the *E. coli* pET28a(+) expression vector for simulation.

### 2.10 Immune simulation analysis

C-ImmSim online web server (121, 122) was employed for the immunogenic profiling of the translated vaccine sequence. This online server used machine learning algorithms and PSSM (position-specific scoring matrices) to access virtual immune reactions in the human body. According to standard practice, we administered three doses of 1000 molecules of antigens with a four-week gap in the virtual human system. After the first dose of vaccine administration, we followed the administration of the second and third doses at the eighth and 24th weeks, with 168 and 504 steps, respectively. The first-time step for initial injection at t=0 corresponds to 8 hours in real time for each time step. This immune simulation was conducted over 1050 simulation steps, with the remaining parameters set to their default values (109, 123).

## 3 Results

### 3.1 Protein sequence retrieval and multiple sequence alignment

The conserved segments of HIV-1 enveloped gp120 sequences were obtained after a multiple-sequence alignment process to advance vaccine development. Targeting conserved epitopes in vaccine development refers to focusing on antigenic regions that are less variable and more stable, which could potentially provide a more universal vaccine approach. This strategy aims to address the challenges posed by the high variability and immune evasion mechanisms of HIV. By targeting conserved epitopes, researchers can develop vaccines that are more effective in eliciting protective immune responses and reduce the risk of vaccine failure due to antigenic variation (124–126). The absence of highly conserved regions in HIV-1 gp120 presents both challenges and opportunities for the development of innovative vaccine strategies. Targeting specific vulnerable sites within variable regions and combining conserved and selected epitopes aims to enhance immune responses. Advances in structural biology and bioinformatics will help design immunogens to activate bnAb-producing B cell lineages, advancing HIV-1 vaccine development (42, 127, 128).

### 3.2 Antigenicity and physicochemical properties of the gp120 protein

The protein candidate for designing a vaccine conjugate must be antigenic to trigger the cellular and humoral immune response. In this respect, the VaxiJen v2.0 server validated the antigenicity of the HIV-1 conserved gp120 sequence, achieving a score of 0.5804. In the next step, the online ProtParam tool was used to calculate the physicochemical properties of gp120, which revealed the theoretical pI in the basic pH range (**Table 1**).

**Table 1:** Physiochemical properties of HIV-1 gp120 sequence predicted by ProtParam.

Physiochemical properties	gp120
Molecular weight (kDa)	51.08
Antigenicity	0.58
Amino acids number	455
Theoretical pI	8.53
Ec (M^-1^ cm^-1^, at 280nm)	53035
Estimated half-life	>10 hours (*E. coli*, *in vivo*), >20 hours (yeast, *in vivo*), and 100 hours (mammalian reticulocytes, *in vitro*)

### 3.3 Epitopes prediction and important features profiling

We employed the IEDB server to predict MHC-I, MHC-II, and B-cell binding epitopes within the conserved HIV-1 gp120 sequence. Specifically, the NetMHCpan EL 4.1 method was utilized for MHC-I binding epitope prediction, while the IEDB recommended 2.22 method was employed for MHC-II binding epitope prediction. Additionally, B-cell epitopes were forecasted using the BepiPred Linear Epitope Prediction 2.0 method. Subsequently, we systematically organized all identified MHC-I, MHC-II, and B-cell binding epitopes. To ensure consistency and efficacy, we refined MHC-I, MHC-II, and B-cell binding epitopes by selecting conserved, antigenic, non-allergenic, and non-toxic, as presented in **Tables 2**–**4**. Population coverage is crucial in vaccine development, representing the percentage of individuals requiring vaccination to achieve herd immunity. The greater the population coverage, the more influential the vaccine is in preventing viral transmission. Therefore, we assessed the population coverage of MHC-I and MHC-II epitopes specifically within the South Asian population, encompassing India and Pakistan, utilizing IEDB’s Population Coverage tool (**Tables 2**, **3**). The following important factor, epitope conservancy, is vital for identifying HIV-1 gp120 conserved epitopes. This enables vaccine developers to design vaccines that are effective against multiple HIV-1 strains, thereby enhancing their breadth of coverage. In the present study, the epitope conservancy analysis validated the preservation of all chosen MHC-I, MHC-II, and B-cell epitopes, reaffirming their suitability for developing a gp120 HIV-1 vaccine.

**Table 2:** Antigenic MHC-I binding epitopes of HIV-1 gp120 predicted by IEDB server.

Peptides	Position	Antigenicity score	Toxicity	Allergenicity	Population coverage
QQKVHALFY	131-139	0.6453	No	No	94.73%
ITIGPGQVF	270-278	0.6102	No	No	94.73%
WQGVGQAMY	373-381	0.5972	No	No	94.73%
APTRAKRRV	441-449	0.9926	No	No	94.73%

**Table 3:** Antigenic MHC-II binding epitopes of gp120 of HIV-1 predicted by the IEDB-recommended 2.22 method using the IEDB server.

Peptides	Position	Antigenicity score	Toxicity	Allergenicity	Population coverage
KQQKVHALFYRLDIV	130-144	0.8393	No	No	75.38%
QQKVHALFYRLDIVQ	131-145	0.5084	No	No	75.38%
QKVHALFYRLDIVQI	132-146	0.6249	No	No	69.90%
SLAEEEIIIRSENLT	227-241	0.6907	No	No	75.38%
IRSENLTNNVKTIIV	235-249	0.469	No	No	75.38%

**Table 4:** Antigenic B-cell epitopes of gp120 from HIV-1 were predicted using the BepiPred linear epitope prediction 2.0 method on the IEDB server.

Peptide	Position	Length	Antigenicity Score	Antigenicity	Allergenicity
PNPQEI	38-43	6	1.2929	Yes	Yes
LENVTENFNMWKNN	45-58	14	-0.268	No	No
NDKNFNGTGPCKNVSS	192-207	16	0.4699	Yes	Yes
SNNTRT	263-268	6	0.4082	Yes	Yes
DIIGDI	283-288	6	-0.9179	No	Yes
KQIINMWQGV	367-376	10	-0.4973	No	Yes
IEPLGIAPTRAKRRVVER*	435-452	18	1.003	Yes	No

*The B-cell epitope selected for vaccine designing.

### 3.4 Vaccine construction, characterization, and structures prediction

**Figure 1:**
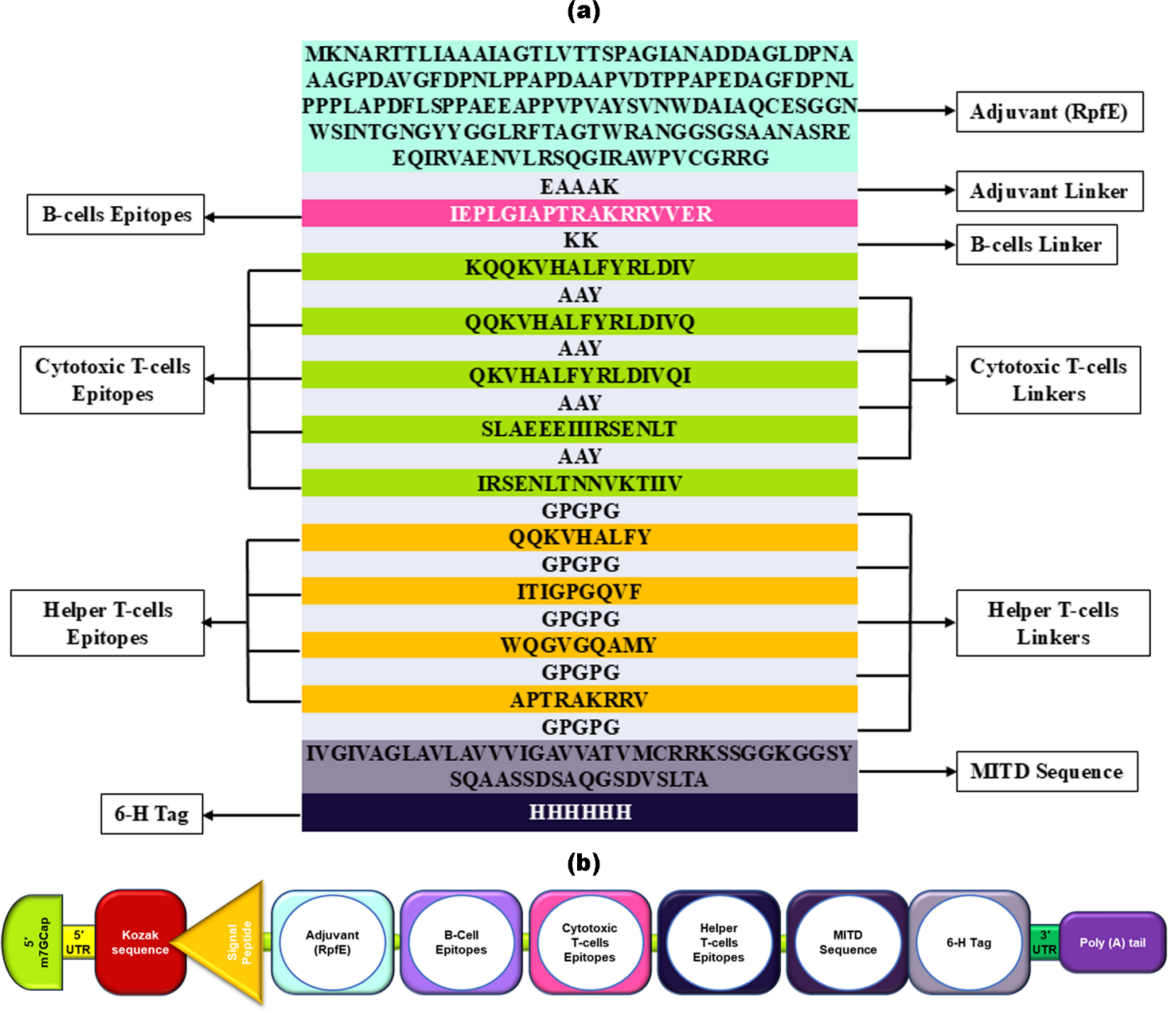
Illustrates the structural arrangement of the HIV-1 gp120 vaccine candidate. **(a)** The arrangement of the gp120 vaccine, showing its amino acids starting from an adjuvant, B-cell, CTL (cytotoxic T-cell), and HTL (helper T-cell) epitopes, each separated by linkers of a few amino acid sequences, followed by MITD and a 6-His tag. **(b)** schematic and simplified representation of mRNA HIV-1 gp120 vaccine components.

The vaccine design commenced at the N-terminus, incorporating a RpfE adjuvant connected with the B-cell epitope via the EAAAK linker. RpfE is an agonist of the TLR4 receptor that enhances, accelerates, and prolongs the specific immune response with a boost in the production of Abs (129). The HIV-1 vaccine includes one B-cell epitope, five MHC-I epitopes, and four MHC-II epitopes, eliciting immune responses specific to the gp120 antigen. The vaccine construct incorporates a concluding vaccine sequence at the C-terminal end, including the MITD sequence and 6x His tag (**Figure 1A**). This particular sequence directs peptides to specific areas of the endoplasmic reticulum and Golgi apparatus, facilitating the effective secretion and presentation by MHC-I and MHC-II. (80). Based on proteins, the vaccine was initially converted into an RNA sequence through reverse translation. Subsequently, the sequence was optimized using JCAT. Following this, the mRNA vaccine construct for HIV-1 gp120 started from the N-terminal with 5′ m7GCap–5′ UTR–Kozak sequence–Signal peptide (tPA)–EAAAK linker- Adjuvant (RpfE)—EAAAK Linker–IEPLGIAPTRAKRRVVER–KK Linker–KQQKVHALFYRLDIV–AAY Linker–QQKVHALFYRLDIVQ–AAY Linker–QKVHALFYRLDIVQI–AAY Linker–SLAEEEIIIRSENLT–AAY Linker–IRSENLTNNVKTIIV–GPGPG Linker–QQKVHALFY–GPGPG Linker–ITIGPGQVF–GPGPG Linker–WQGVGQAMY–GPGPG Linker-APTRAKRRV–GPGPG–MITD sequence–6-H tag–Stop codon–3′ UTR–Poly (A) tail at the C-terminal, as depicted in **Figure 1B**. The mRNA HIV-1 gp120 vaccine sequence is shown in **Supplementary Table S1**.

**Figure 2:**
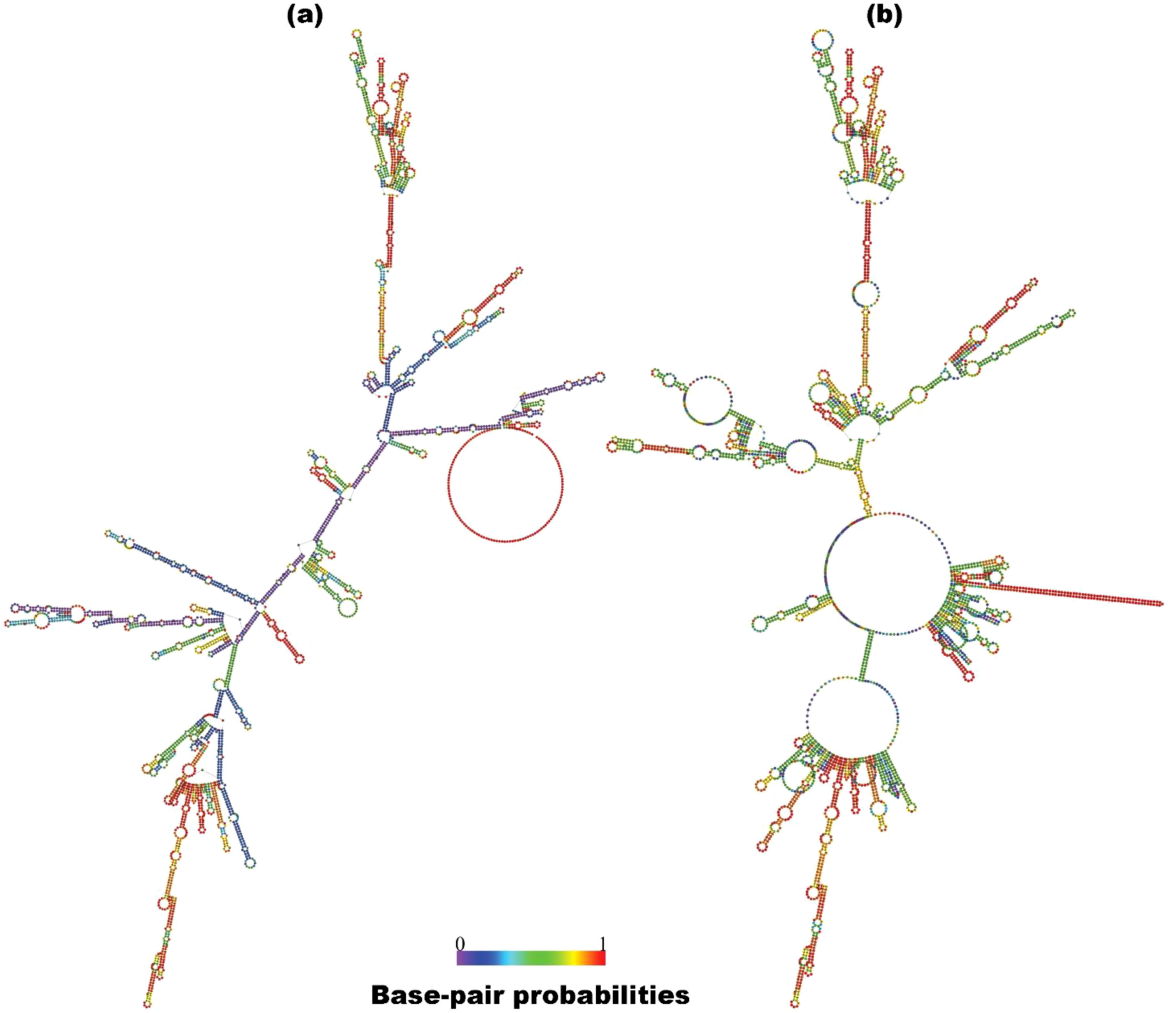
Predicted MFE **(a)** and centroid **(b)** secondary structure of mRNA features of the HIV-1 gp120 vaccine with base-pair probabilities.

The mRNA HIV-1 gp120 vaccine demonstrated an optimal secondary structure with a minimal free energy of -1463.30 kcal/mol, while the centroid secondary structure exhibited a minimal free energy of -1125.55 kcal/mol. Additionally, the thermodynamic free energy of the mRNA HIV-1 gp120 vaccine was determined to be -1509.60 kcal/mol, with the MFE structure occurring at a frequency of 0% and an ensemble diversity of 949.07. **Figure 2** shows the MFE and Centroid secondary structures of the mRNA HIV-1 gp120 vaccine.

After entering the host’s immune cells, the mRNA vaccine gets translated by the ribosomes into antigenic proteins that initiate an adaptive immune response, kickstart innate immunity, promote Ab production, and elicit long-term cellular immunity, providing new options for traditional vaccines (89, 90). The molecular characteristics of the translated protein-based HIV-1 gp120 vaccine exhibit unique physicochemical attributes tailored to their specific target, as shown in **Supplementary Table S2**. The vaccine has the same theoretical pI as the gp120 protein (9.14 for the vaccine construct and 8.53 for the gp120 protein), indicating a possible link between stability and immunogenicity. This highlights the significance of factoring in the pI when designing the vaccine construct (130, 131). Additionally, the vaccine’s antigenicity was validated using the VaxiJen 2.0 server, which yielded an antigenicity score of 0.63, and ANTIGENpro provided a score of 0.93. Furthermore, to ensure suitability, AllerTop 2.0 and Toxinpred2 assessments supported the non-allergenicity and non-toxicity of the HIV-1 vaccine construct. Notably, the vaccine’s solubility score exceeded the threshold value of 0.5, registering 0.697, which affirmed its potential for insoluble expression in *E. coli* ( **Supplementary Table S2**).

**Figure 3:**
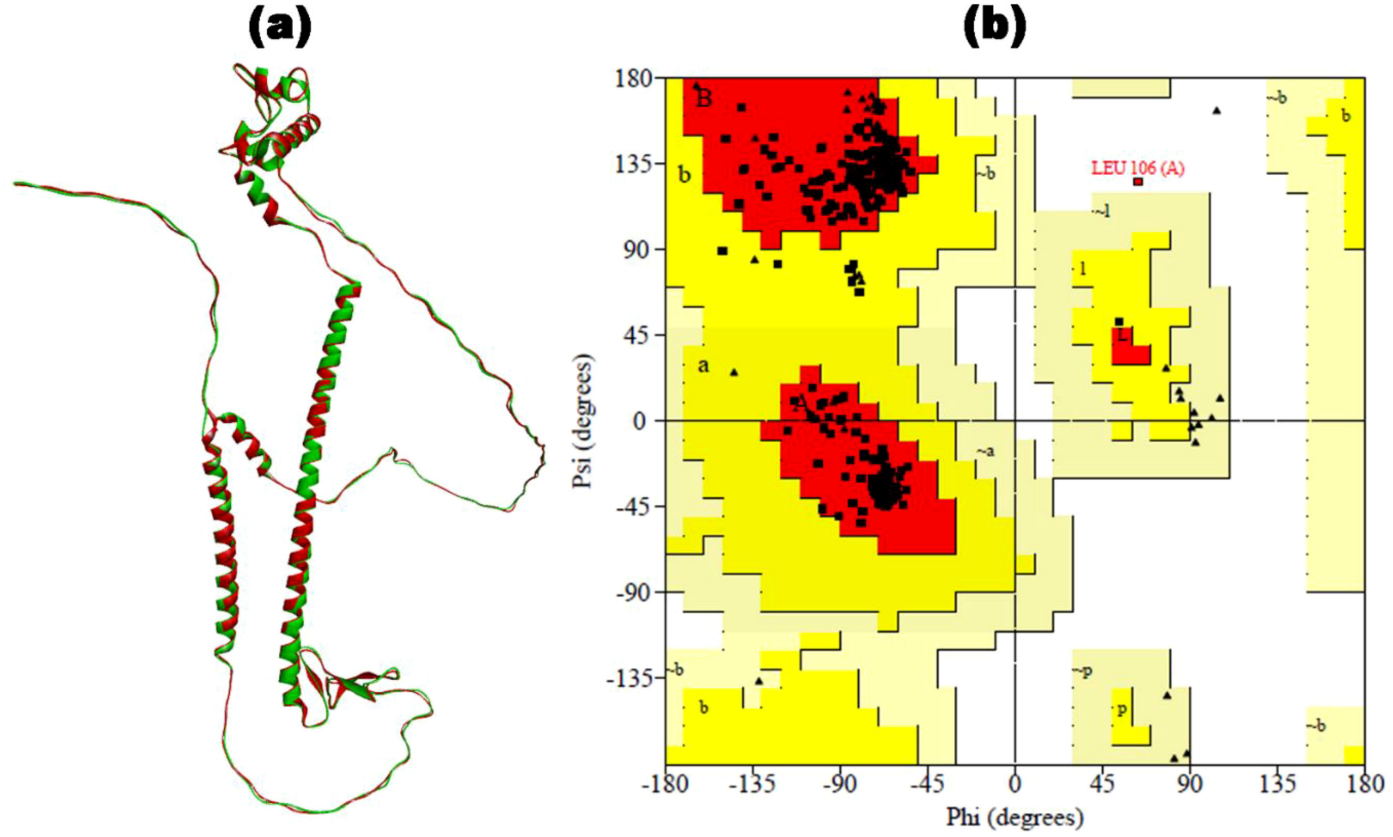
The 3D structure of the HIV-1 gp120 vaccine was constructed. **(a)** The 3D structure predicted by ColabFold (in green) is superimposed by the refined 3D structure of the HIV-1 gp120 vaccine by GalaxyRefine (in red). **(b)** Ramachandran plot of refined gp120 vaccine construct with 97.4% of residues in the most favored region.

The SOPMA web server computed the secondary structure elements, i.e., alpha helix (15.02%), extended strand (13.79%), and random coil (71.18) within the translated HIV-1 gp120 vaccine. The PSIPRED results provide insights into the presence of the predicted secondary structure elements in the vaccine construct ( **Supplementary Figure S1**). In the next step, we obtained five distinct 3D models of the vaccine by ColabFold. Each model is associated with a C-score ranging from -5 (indicating poor quality) to 2 (indicating good quality). The final representative 3D structure with the maximum C-score value for the HIV-1 gp120 vaccine is shown in **Figure 3A** (in green). The Ramachandran plot confirmed 57.3% of the residues falling within the most favored regions in the 3D structure of the HIV-1 gp120 vaccine. However, a high-quality model would typically have over 90% of the residues in these regions. Consequently, refinement is necessary for our predicted 3D structure of the HIV-1 gp120 vaccine.

### 3.5 Refinement and verification of 3D vaccine

The 3D structure of the HIV-1 gp120 vaccine was meticulously enhanced by applying MDS for refinement using the online web server GalaxyRefine. Five refined 3D structures of the HIV-1 gp120 vaccine were obtained, out of which the region with the maximum Rama-favored score was selected for further analysis. The refined 3D structure of the HIV-1 gp120 vaccine with 99.1% Rama-favored region is depicted in **Figure 3A** (in red). Subsequently, the refined 3D vaccine structure was rigorously assessed using a Ramachandran plot (**Figure 3B**). In the refined 3D model context, 97.2% of residues were found within the most favored region of the Ramachandran plot, indicating a well-constructed model. Furthermore, the plot offered insightful information on several key parameters, including residues within the additionally allowed region (2.5%), end residues (excluding glycine and proline) (2), glycine residues (47), and proline residues (36). Notably, the proportion of residues positioned in the outlier region of the Rama plot was minimal, standing at only 0.3%. The single outlier residue was Leu106, present in the adjuvant region.

### 3.6 Linear and discontinuous B-cell epitope prediction of vaccine construct

**Figure 4:**
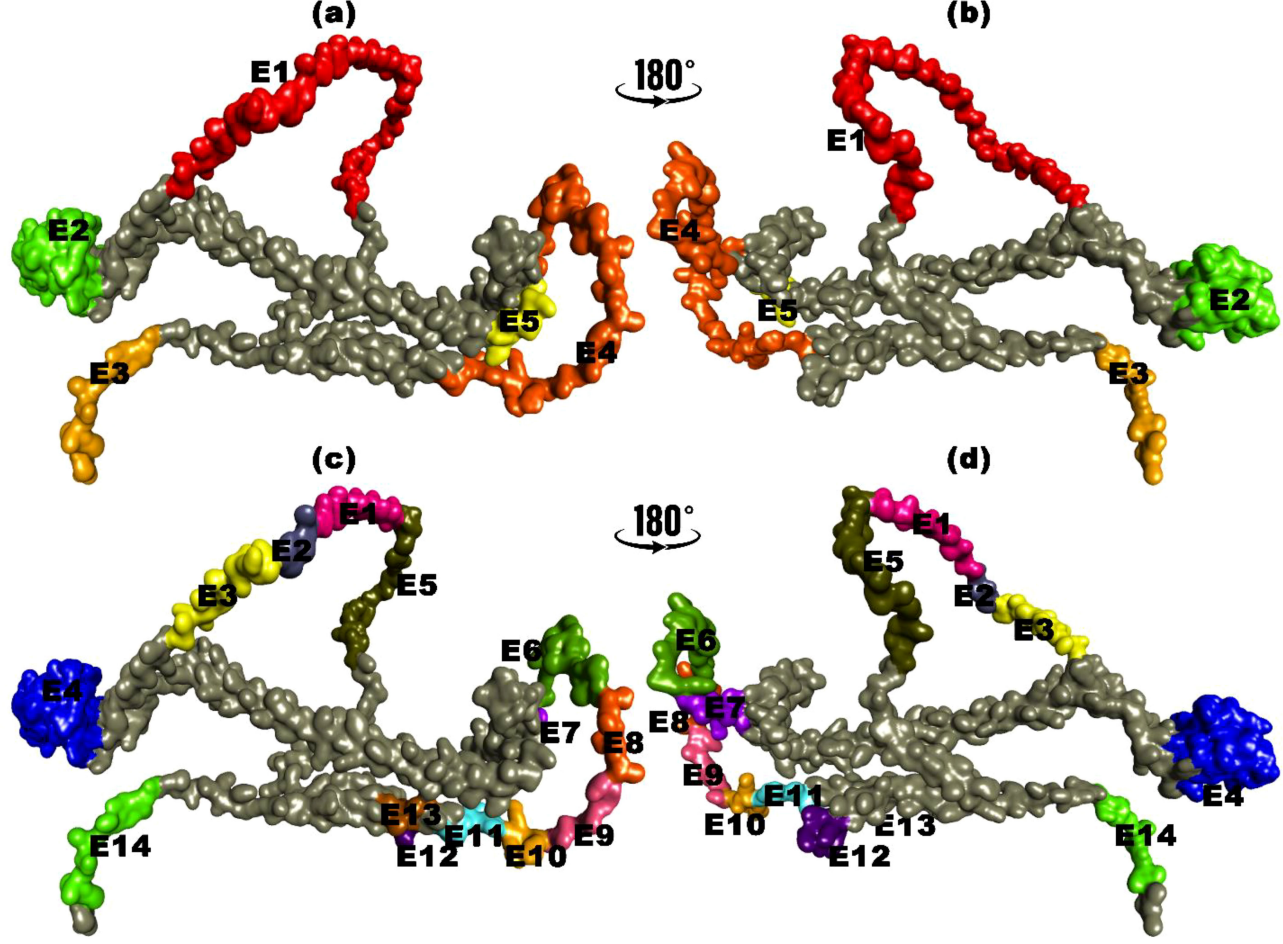
B-cell epitopes mapping for translated HIV-1 gp120 vaccine. **(a, b)** Linear B-cell epitopes with amino acid residues. **(c, d)** Discontinuous B-cell epitopes with amino acid residues.

Including substantial B-cell epitopes within the vaccine is paramount as it is crucial in triggering humoral immunity and producing Abs and cytokines against foreign antigens. The ElliPro tool was utilized to ascertain the presence of these essential elements, revealing the existence of five linear B-cell epitopes within the vaccine. These epitopes spanned amino acid residues 8 to 68 and exhibited PI scores ranging from 0.54 to 0.844. Concurrently, the vaccine contained 14 discontinuous B-cell epitopes, with residues ranging from 4 to 67, all exhibiting PI score values between 0.54 and 0.94. A graphical representation of these epitopes is shown in **Figure 4**.

### 3.7 Molecular docking and molecular dynamic simulation

**Figure 5:**
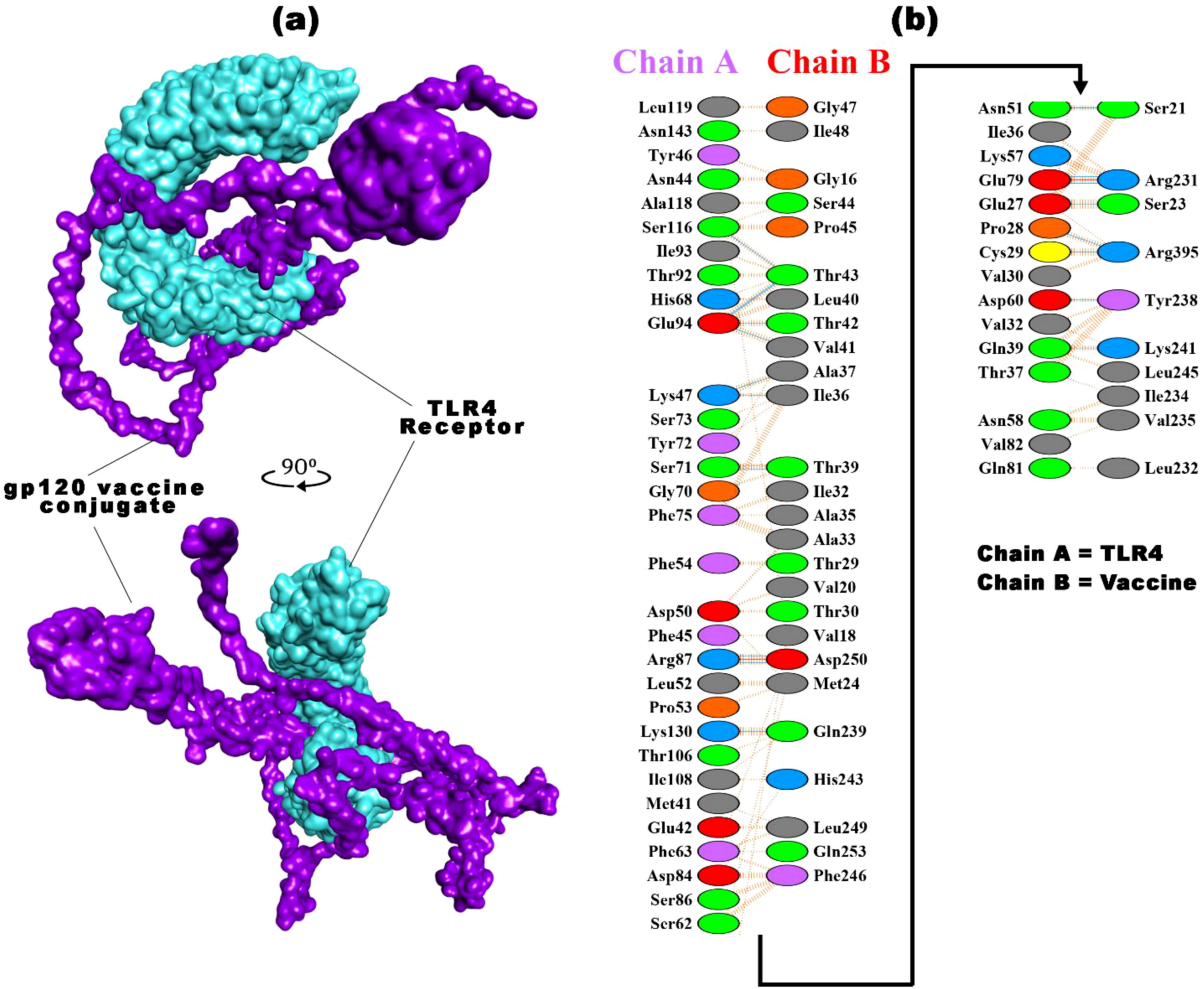
The best vaccine-TLR4 docked result was obtained from ClusPro 2.0. **(a)** The docked complex of the HIV-1 gp120 vaccine construct with the TLR4 receptor **(b)** highlights the interacting residues of TLR4 (chain A) with the vaccine (chain B), along with the types of bonds.

The vaccine-TLR4 docking process assessed the binding affinity among 30 complexes obtained from ClusPro 2.0. The vaccine bound with the Toll/interleukin-1 receptor (TIR) domain of TLR4 protein, with a binding affinity of -1181.2 kcal/mol, was selected for detailed analysis. The TIR domain is recognized for its pivotal role in TLR4 signaling, located in the intracellular region of the receptor, which facilitates the binding of diverse adaptor proteins and triggers intracellular signaling cascades (132). This complex was visualized using PyMol, Discovery Studio, and pdbsum (**Figure 5A**), forming 39 hydrogen bonds and 514 non-bonded contacts at their interfaces. TLR4 recognizes the vaccine’s pathogen-associated molecular patterns (PAMPs) and boosts the immune response. Upon binding to TLR4, a vaccine initiates a signaling cascade that involves the recruitment of adaptor proteins (e.g., MyD88 and TRIF) and the activation of kinases (e.g., IRAK and TBK1). Subsequently, these kinases phosphorylate transcription factors, such as NF-κB and IRF3, facilitating their translocation to the nucleus and inducing the expression of pro-inflammatory cytokines and interferons. This molecular response enhances the adaptive immune response, facilitating the generation of vaccine-specific Antibodies and memory cells (107, 133). Different experimental studies have verified the activation of TLR4 by various viral glycoproteins. TLR4 is crucial for NF-κB activation by EBOV GP, and blocking TLR4 activity prevents the release of cytokines and other immune responses. However, the role of TLR4 in RSV infection remains uncertain and appears variable based on the specific mouse strain involved (134–137).

The comprehensive summary of the bonding network and other essential parameters are mentioned in **Figure 5B** and **Supplementary Table S3**. The TLR4-vaccine complex exhibits a strong interaction involving 46-36 interface residues and an expansive interface area of 1,847-1,993 Å². The binding affinity is calculated to be -1181.2 kcal/mol, with significant contributions from electrostatic and hydrophobic forces. The combined van der Waals and electrostatic binding affinity is -322.7 kcal/mol. Notably, the complex forms two salt bridges and 20 hydrogen bonds, exhibiting 223 non-bonded contacts, which underscores a well-established and diverse molecular association. These docking parameters collectively emphasize the robust and intricate nature of the binding between TLR4 and the gp120 vaccine. Docking analysis of vaccine with TLR4 computationally predicts the immunogenicity and efficacy of vaccine candidates. These approaches have limitations because they rely on the availability and accuracy of data for target molecules, which may be incomplete or outdated. They may inadequately capture complex immune interactions, neglecting key factors such as antigen processing, presentation, and recognition. They also overlook host population variability and practical aspects of vaccine development. Despite their value in screening, in silico tools cannot replace experimental validation and clinical trials for confirming vaccine efficacy (138, 139).

**Figure 6:**
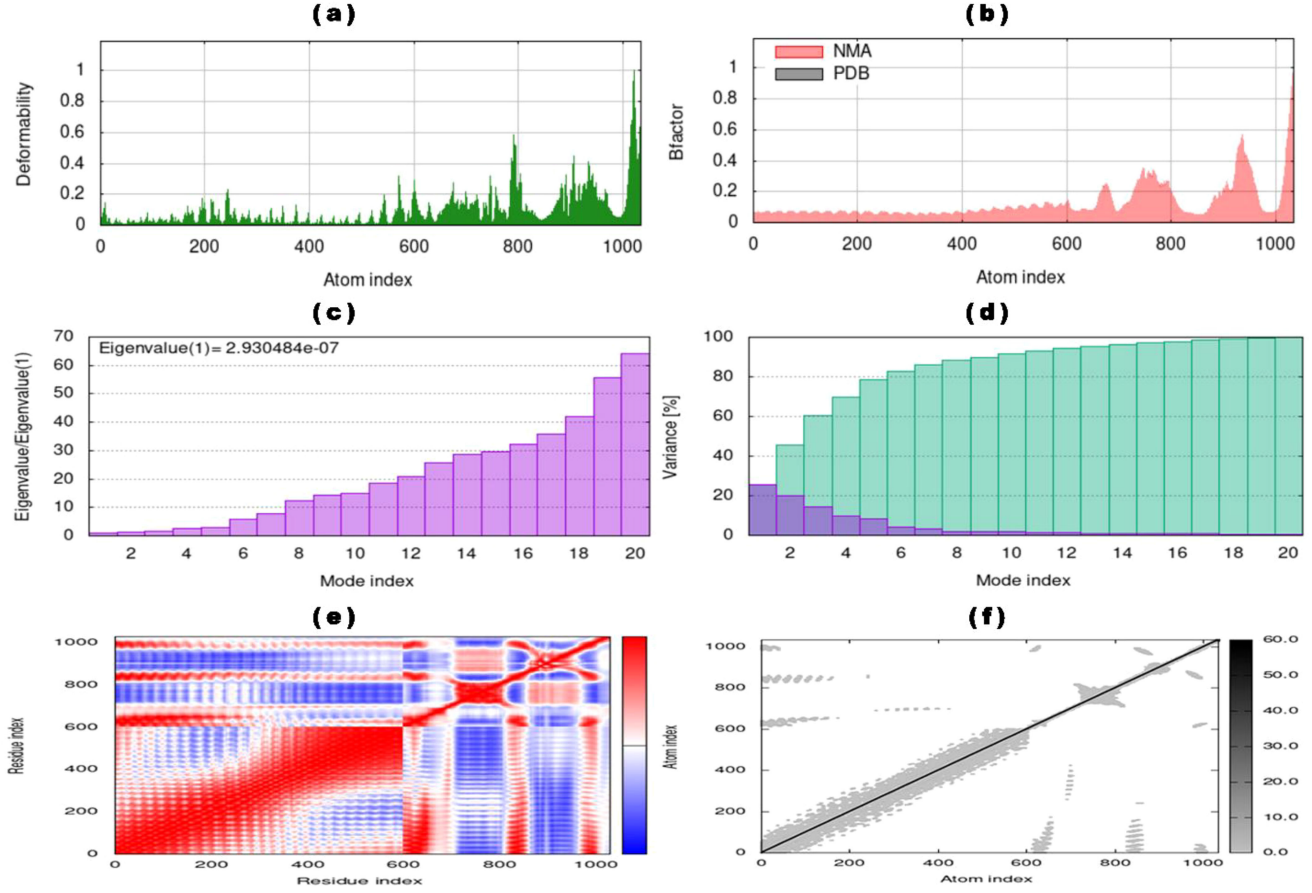
NMA of vaccine-TLR4 docked complex determined by iMODs server **(a)** Deformability of vaccine-TLR4 complex with atom index, **(b)** B-factor of the docked complex representing the NMA result compared with atom index, **(c)** eigenvalue with mode index, **(d)** percentage variance (individual variances showing in violet color and cumulative variances showing cyan color) of the vaccine-TLR4 complex with mode index, **(e)** co-variance map of the vaccine-TLR4 complex for residue index (anti-correlated motions showing blue, correlated motions showing red, and uncorrelated motions showing white), and **(f)** elastic network map with atomic index (stiffer regions showing in darker gray regions).

Subsequently, the iMODS tool analyzed the stability and conformational dynamics of the vaccine-TLR4 docked complex. iMODS evaluates the stability, physical movements, and internal coordinates of the vaccine-receptor complex. This enables the assessment of the host immune system’s response to the vaccine candidate. Particularly advantageous for in-silico vaccine design, iMODS provides a swift and cost-effective alternative to conventional experimental methodologies (140–142). The atomic index deformability of the vaccine-TLR4 complex is visualized in **Figure 6A**, offering insights into the deformability of specific regions. Notably, the ending residues show higher deformability values corresponding to chain hinges. The dynamicity of the docked complex, as indicated by the B-factor values derived from NMA, is directly related to the root mean square (RMS) value, as shown in **Figure 6B**. The eigenvalue of the vaccine-TLR4 docked complex, quantified as 2.930484 x 10^-7^ and depicted in **Figure 6C**, signifies the rigidity of the complex. A lower eigenvalue suggests greater susceptibility to deformation. The variance graph, displayed in **Figure 6D**, illustrates the proportional contributions of individual normal modes to the equilibrium movements. Cyan bars indicate combined variance, while violet bars represent individual variance. Furthermore, the covariance graph in **Figure 6E** outlines the mobility patterns within distinct molecular regions, with red, blue, and white colors denoting correlated, uncorrelated, and anti-correlated movements, respectively. Lastly, the elastic network graph in **Figure 6F** illustrates the springs connecting atom pairs, with each dot representing a spring and a darker gray dot indicating stiffer springs.

**Figure 7:**
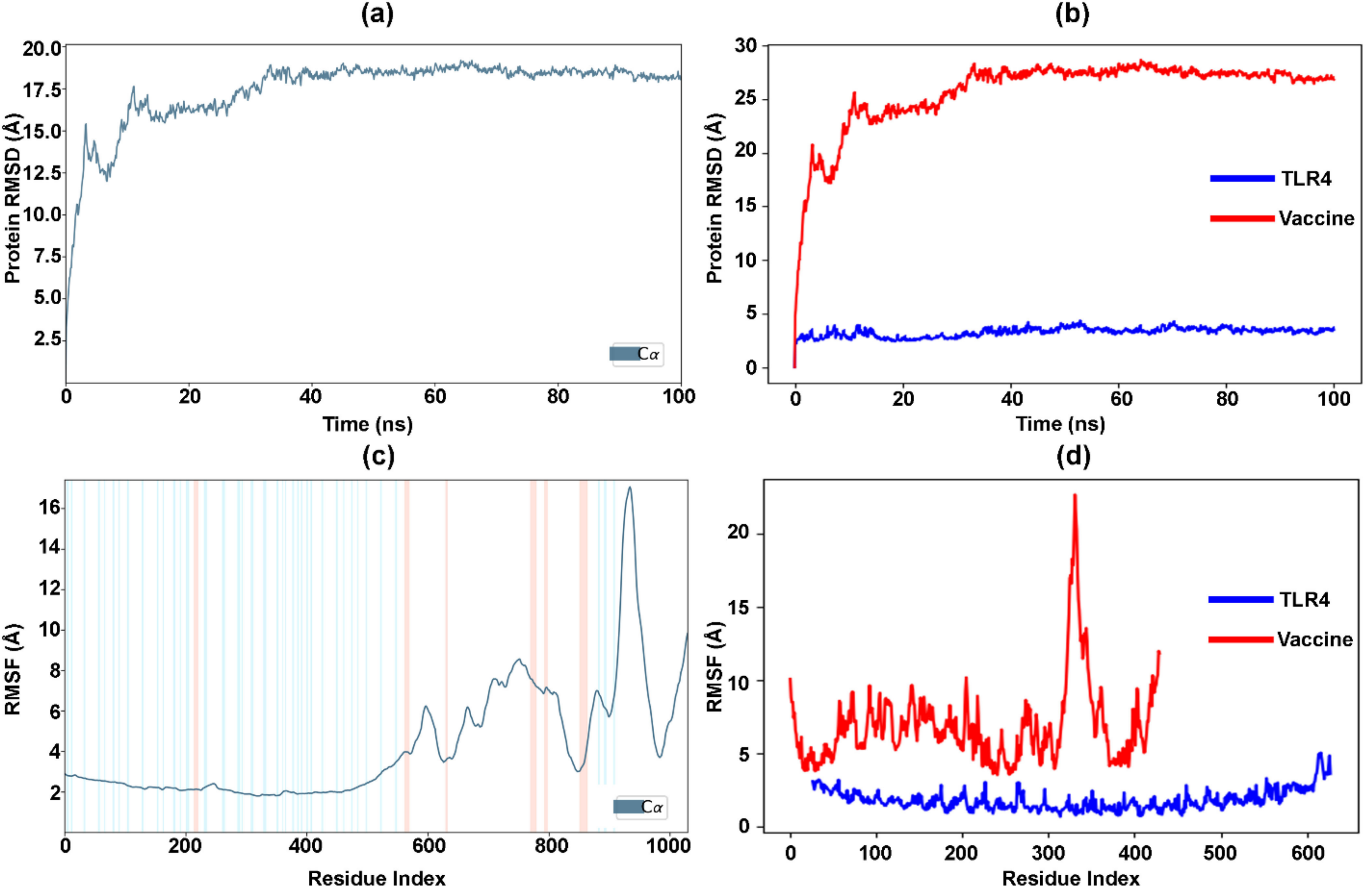
Analysis of vaccine-TLR4 interactions over 100 ns simulation. **(a)** RMSD changes for C-alpha atoms in the vaccine-TLR4 complex. **(b)** RMSD of separated vaccine and TLR4. **(c)** RMSF values in the vaccine-TLR4 complex. **(d)** RMSF values of separated vaccine and TLR4.

Using MDS, the optimal vaccine-TLR4 interactions were simulated for 100 ns through Desmond, which analyzed the trajectories. The time-dependent changes in RMSD values for the C-alpha atoms in vaccine-TLR4 interactions are depicted in **Figure 7A**. The RMSD plot demonstrates that the vaccine-TLR4 interactions stabilized after 40 ns and remained stable throughout the interval, with the RMSD remaining within the range of approximately 17.5–19 Å. Similarly, the RMSD values of the separated vaccine and TLR4 (**Figure 7B**) showed that the TLR4 receptor became stable at the start of the simulation. In contrast, the vaccine followed the same stability pattern as the vaccine-TLR4 complex after 40 ns. The RMSD of TLR4 remained consistently low, within the range of approximately 1.5–3.5 Å, while the vaccine’s RMSD remained approximately 25–29 Å.

Furthermore, **Figure 7C** displays the RMSF values of the vaccine-TLR4 docked complex. The residue index with more extraordinary peaks is found in the C-terminal zone, starting from 600, as observed in the MD trajectories. The RMSF values exhibit a peak pattern similar to that of the NMA graph (**Figure 6B**). The low and less fluctuating RMSF values of TLR4 indicate the stability of the receptor in the docked complex (**Figure 7D**). Conversely, the high fluctuating peaks at the C-terminal zone of the vaccine indicate higher flexibility and potential interaction with TLR4. Similarly, **Supplementary Figure S2A** represents the number of hydrogen bonds between the vaccine and the TLR4-docked complex during the simulation. The hydrogen bonds increased with the time interval, confirming the stability of the docked complex over time. Furthermore, the percentage of the docked complex’s secondary structure elements (SSE) with respect to the time interval and residue index was 22.04%, comprising 8.7% helix (peaks in red) and 13.34% strand (peaks in blue) ( **Supplementary Figure S2B-C**).

### 3.8 Molecular mechanics and generalized born surface area calculations

**Figure 8:**
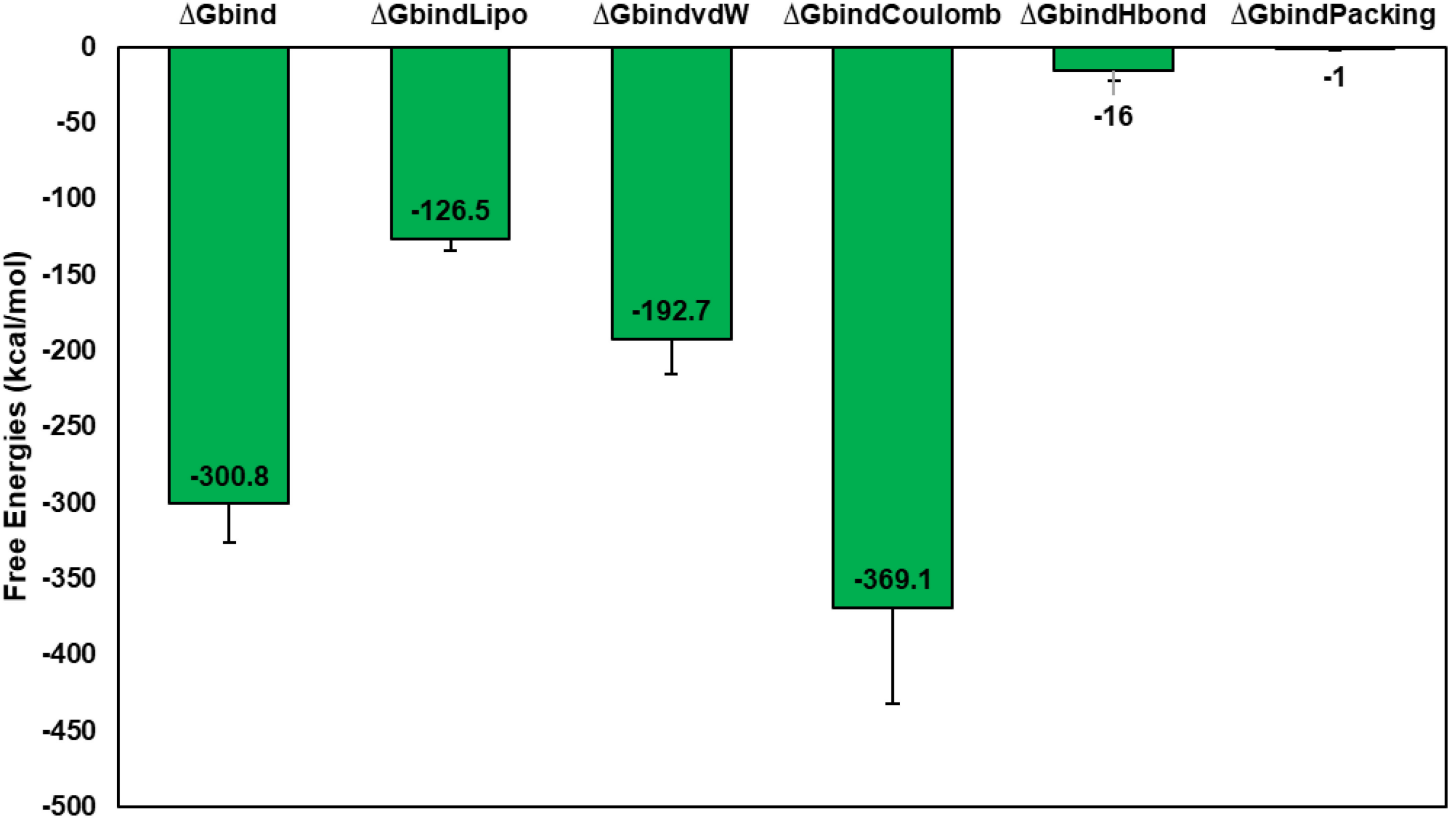
Calculation of average MM-GBSA binding energies, i.e., G_bind_, G_bind_Coulomb, G_bind_Packing, G_bind_H_bond_, G_bind_Lipo, and G_bind_vdW of Vaccine-TLR4 complex from MDS trajectories.

The MM-GBSA method is commonly used to evaluate the binding energy of a vaccine to the TLR4 receptor (143). Our study assessed the impact of additional non-bonded interaction energies and ΔG_bind_ for the vaccine-TLR4 complex. Overall, the ΔG_bind_ of the Vaccine-TLR4 complex was determined to be -300.8 ± 25.34 kcal/mol. We utilized the G_bind_ metric to analyze non-bonded interactions, including G_bind_Coulomb, G_bind_Packing, G_bind_H_bond_, G_bind_Lipo, and G_bind_vdW (**Figure 8**). Our analysis indicated that the average binding energy was predominantly influenced by G_bind_vdW (-192.7 ± 22.22 kcal/mol), G_bind_Lipo (-126.5 ± 7.41 kcal/mol), and G_bind_Coulomb (-369.1 ± 63.13 kcal/mol) energies across all interaction types. Furthermore, stable hydrogen bonds were observed between the vaccine-TLR4 complex and amino acid residues, as evidenced by their G_bind_H_bond_ interaction value of -16 ± 6.41 kcal/mol. These results support the validity of the MM-GBSA calculations derived from the MDS trajectories and align with the binding energy trends obtained from docking data (144).

### 3.9 Codon optimization and *in-silico* cloning

**Figure 9:**
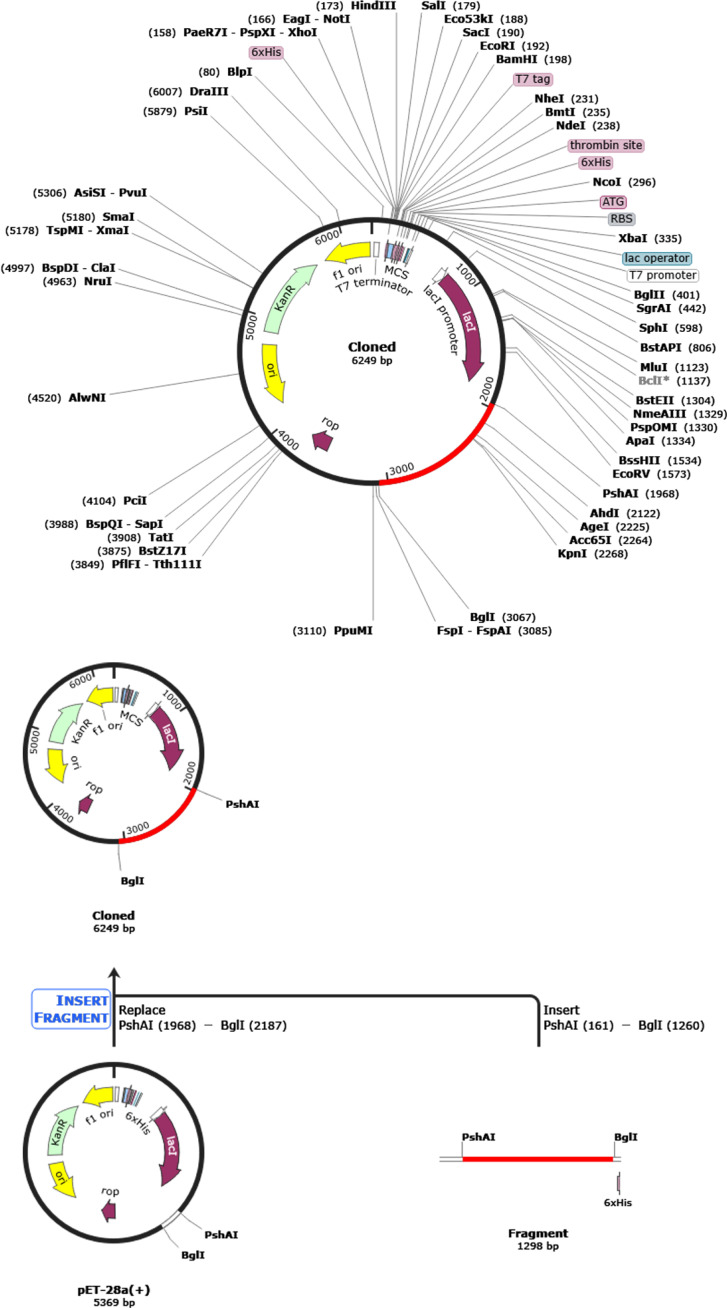
Creating a new and enhanced vaccine and integrating it into the *E. coli* expression vector pET-28a (+) for digital cloning using SnapGene 4.2 software. The gene of interest, located between PshAI (1968) and BglI (3067), is shown in red, and the expression vector pET-28a (+) is depicted in black.

Codon optimization in gene cloning involves selecting host organism-preferred codons for a cloned gene, enhancing expression, avoiding irregular codons, and reducing secondary structure formation. It helps improve protein folding, mitigate toxicity, and streamline the cloning process by ensuring compatibility with the host’s translation machinery. This process ultimately enhances successful gene expression in the host organism (145, 146) (147). The JCat tool was used to fine-tune the vaccine sequence, which was tailored explicitly for the E. coli K-12 strain, resulting in a sequence with a GC content of 56.17%. Due to the basic translation machinery in bacteria, this optimization validates the efficacy of high-yield vaccine expression in E. coli bacteria, as underscored by a Codon Adaptation Index (CAI) value of 1.0. After this refinement, the enhanced and optimized DNA sequence of the vaccine construct was incorporated into the *E. coli* expression vector PET28a(+), strategically positioned between the PshAI (161) and BglI (1260) restriction sites, as depicted in **Figure 9**. This construction yielded a clone with a length of 6.2 kilobase pairs. Following the overexpression of the vaccine protein in E. coli, the recombinant vaccine underwent purification via affinity chromatography, facilitated by the inclusion of a 6-histidine tag at the terminus.

### 3.10 Immune simulation analysis

**Figure 10:**
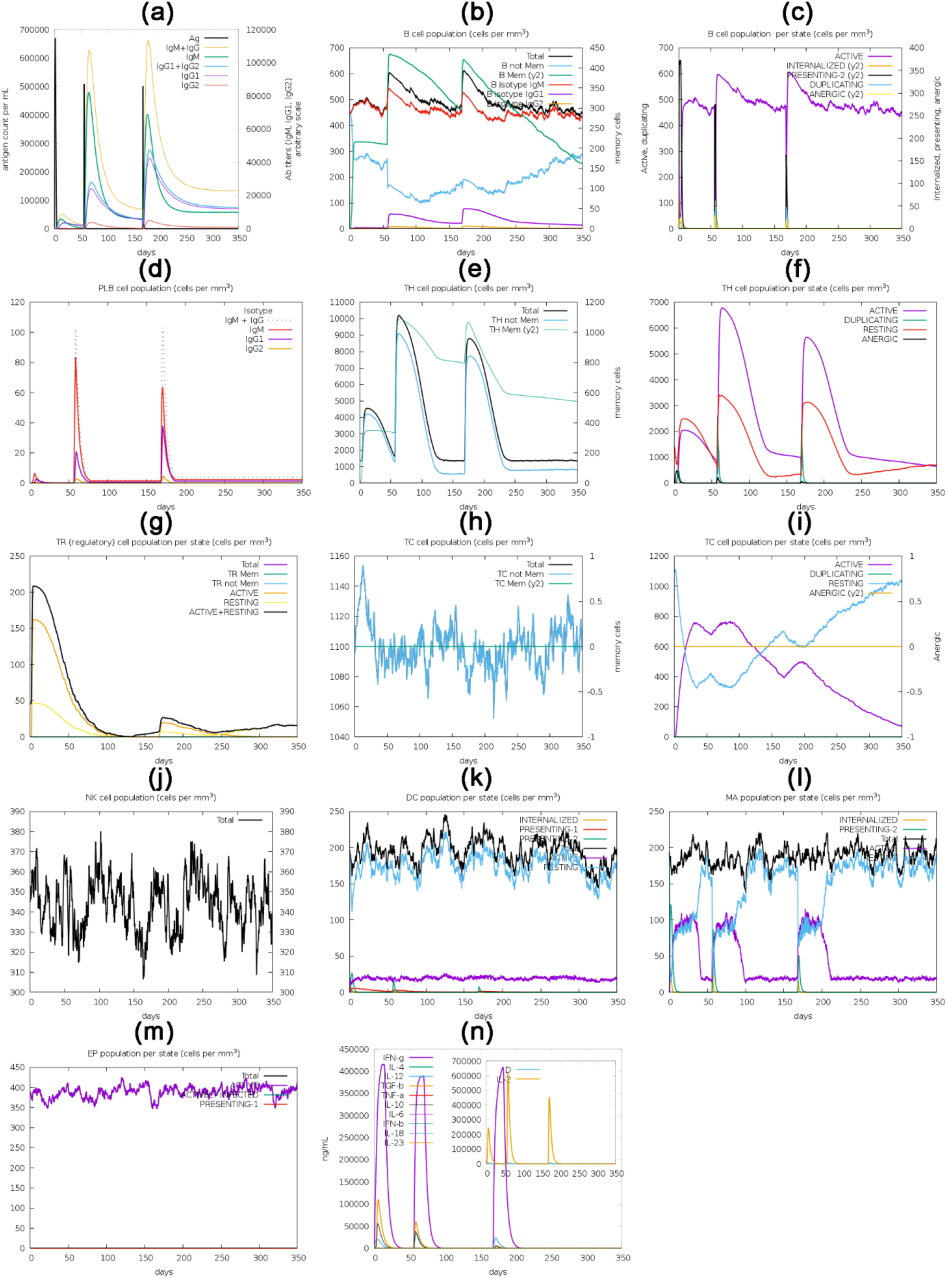
The translated HIV-1 gp120 vaccine, used as an antigen, was administered via three injections over 350 days for in silico immune response simulation. The simulation evaluated multiple parameters, such as the **(a)** immunoglobulins and antigen, **(b)** B-cell population, **(c)** B-cell population per state (cells/mm^3^), **(d)** plasma B-cell population (cells/mm^3^), **(e)** helper T-cell population (cells/mm^3^), **(f)** helper T-cell population per state (cells/mm^3^), **(g)** Th1 cell population, regulatory T-cell population per state (cells/mm^3^), **(h)** cytotoxic T-cell population (cells/mm^3^), **(i)** cytotoxic T-cell population per state (cells/mm^3^), **(j)** natural killer cell population (cells/mm^3^), **(k)** dendritic cell population per state (cells/mm^3^), **(l)** macrophage population per state (cells/mm^3^), **(m)** epithelial presenting cells population per state (cells/mm^3^), and **(n)** interleukins and cytokine concentration.

*In silico* immune simulation analysis, a cornerstone in vaccine development and immunology, employs computational models to simulate immune system responses. This approach is instrumental in predicting, testing, and optimizing immune-related processes, such as vaccine design, drug development, and disease modeling. (148–150)The computational analysis of the C-ImmSim server has yielded findings of significant importance. It has predicted a robust stimulation of the primary immune response, characterized by a gradual increase in immunoglobulin levels, including IgM, IgG1, IgG2, and IgG, following the virtual administration of the first, second, and third vaccine doses in humans. (151). Initial vaccine inoculation enhanced the concentration of immunoglobulins, which subsequently diminished over time. However, a significant resurgence in immunoglobulin concentration was observed after the third dose. Conversely, antigen concentration declined during and after the second and third vaccine doses, as depicted in **Figure 10A**. The active and total B-cell populations exhibited sustained elevation, as shown in **Figures 10B** and **10C**, respectively. The concentration of plasma B-cells increased initially for several days post-vaccination (**Figure 10D**). Likewise, active total helper T-cells exhibited sustained and elevated levels following vaccination (**Figures 10E, F**). However, the concentration of active and resting helper regulatory T-cells was notably high after the initial vaccine dose but exhibited a progressive reduction over time (**Figure 10G**). The concentration of cytotoxic helper T-cells displayed temporal fluctuations, as illustrated in **Figure 10H**, with their active form decreasing while maintaining a constant anergic state after vaccination, as shown in **Figure 10I**. Additionally, the population of natural killer cells showed variability throughout the vaccination process, as depicted in **Figure 10J**. The concentrations of dendritic cells, macrophages, and epithelial presenting cells were quantified in cells per cubic millimeter and are presented in **Figure 10K-M**. Alongside the activation of various cell types, a diverse range of cytokines and interleukins also exhibited increased concentrations following vaccination, as depicted in **Figure 10N**.

## 4 Discussion

In recent years, a growing trend in vaccine development research has been the adoption of multi-omics approaches. These comprehensive strategies harness advanced bioinformatics and structural biology tools to design vaccines based on epitopes and specific antigenic regions of pathogenic microorganisms. These studies have yielded promising results and constitute a substantial portion of the ongoing research in the field of vaccine development (152, 153). Traditional methods for developing and enhancing various vaccine types, such as live or attenuated vaccines, are costly and labor-intensive. They typically involve extended timelines before reaching commercial availability. Moreover, attenuated vaccines, though moderately effective, provide only limited passive immunity and may trigger allergic reactions owing to their elevated antigenic load (154). These vaccines often offer overly simplified solutions for tackling intricate infectious agents. As a result, scientists have dedicated significant efforts to developing more cost-effective, time-efficient, and safer vaccines in combatting a wide array of infectious diseases. The emergence of multi-omics technology has given rise to the field of immune informatics, offering a promising avenue to confront the challenges associated with vaccine development by creating potent peptide-based vaccines (155–157).

In our study, we set out to develop a vaccine for HIV-1 characterized by hypoallergenic, non-toxicity, and overall safety via artificial intelligence (158–160). The rationale behind choosing HIV as a target for vaccine development is based on the need to develop effective vaccines that can help eliminate HIV as a significant public health problem. A successful HIV vaccine could potentially save millions of lives and reduce the burden on healthcare systems worldwide (161). The vaccine was meticulously crafted by assembling diverse antigenic, non-allergenic, immunogenic, cytokine-inducing, and non-toxic B and T-cell linear epitopes sourced from the HIV-1 envelope gp120 protein. Our study highlights the potential of conserved linear epitopes, which can be enhanced by strategies such as germline-targeting immunogens to prime B cells, sequential immunization to refine antibody maturation, and epitope scaffolding to focus immune responses. These approaches, inspired by recent advances in HIV mRNA vaccine development, offer a promising path for optimizing vaccine design. Subsequently, we subjected the resulting vaccine to comprehensive bioinformatics analysis to assess its interactions with immune system-activating receptors. Additionally, we evaluated its immunogenic potential in real-world scenarios. This research investigated the feasibility of utilizing artificial intelligence in developing advanced HIV-1 vaccines with improved safety and efficacy profiles.

The HIV-1 envelope protein (gp120) plays a vital role by binding to specific receptors on target cells, initiating the virus entry process into host cells. This makes it an essential candidate for vaccine development. The selection of gp120 was also based on its antigenicity score, which stood at 0.58. A higher antigenicity score indicates a greater potential to elicit an immune response (160). Substantial B-cell epitopes within the vaccine construct are paramount. They are crucial in eliciting a robust humoral immune response, ultimately producing Abs and cytokines against foreign antigens (162). Utilizing the ElliPro tool, we identified five linear B-cell epitopes within our vaccine, spanning amino acid residues 8-68, with scores ranging from 0.54 to 0.844. Additionally, we observed the existence of 14 discontinuous B-cell epitopes, encompassing residues 4 to 67, with score values ranging from 0.549 to 0.946, as vividly illustrated in **Supplementary Figure S2**. T cells are vital components of the immune system that control and trigger immune responses. T-cell epitopes are peptide fragments from antigens presented by the MHC molecules of antigen-presenting cells, stimulating effector T cells, memory T cells, and cytokines such as IFN-γ. CTLs induce a cellular immune response, which is crucial for eradicating viruses and infected cells through the recognition of MHC class I. To address HLA polymorphism, we employed A2, A3, and B7 HLA alleles for MHC-I epitope prediction, representing HLA supertypes found in at least 70% of the South Asian population (163–165). To create a vaccine candidate, we predicted potential T and B-cell epitopes utilizing the IEDB and rigorously assessed their suitability. Ultimately, we identified 4 MHC-I epitopes, 5 MHC-II epitopes, and one B-cell epitope for inclusion in the vaccine. These epitopes were meticulously chosen based on a stringent set of criteria encompassing conservancy, interaction with a broad spectrum of HLA alleles, high antigenicity, non-allergenicity, and non-toxicity (158, 159). However, variability in vaccine outcomes attributed to alterations in epitope positions can significantly influence vaccine efficacy and specificity. Our study followed the reported epitopes sequence (166–170). Understanding the impact of epitope positioning on the immune response is crucial for enhancing vaccine design and development, as host factors, genetic variation, and environmental influences can affect vaccine effectiveness (124, 171).

**Supplementary Figure S1** provides a detailed analysis of secondary structure parameters for the HIV-1 gp120 vaccine construct, offering insights into its stability and functionality. Next, we conducted a crucial validation step by assessing the 3D structure of the vaccine through a Ramachandran plot. The Ramachandran plot confirms the high quality of the predicted vaccine structure, with 97.4% of residues in the most favored region. Additionally, it provides insights into other structural elements, including a minimal 0.3% of residues in the disallowed region, reinforcing the vaccine construct’s reliability and suitability for further development and immunogenicity assessment (172). However, the accuracy of the vaccine’s 3D structure is crucial for ensuring the reliability of the vaccine design. Several methodologies are employed for structural analysis, refinement, and validation to achieve this (166, 169). For instance, a computational approach to designing a polyvalent vaccine against the human respiratory syncytial virus involved using the RaptorX online server and homology modeling from the PDB to generate a 3D vaccine model. The vaccine structure was further refined and validated using the Ramachandran plot and z-score analysis, demonstrating a reasonably consistent and stable structure (173). Similarly, the 3D structure of the vaccine against *Echinococcus granulosus* was validated through various analyses, including the Ramachandran plot and Z-score assessment. (174). However, making vaccines is not always straightforward due to the limitations and uncertainties associated with structural analysis and refinement in vaccine design. Structural vaccinology tries to create effective vaccines by changing their building blocks through reverse molecular engineering. This involves modifying the vaccine’s components and testing it in animals to ensure it’s safe and works well (175). The natural complexities of how the vaccine triggers the immune system, along with the numerous tasks involved in vaccine development, introduce uncertainties and risks. Variables like how well a vaccine works can vary, making vaccine design more complicated (176). Although we can now figure out the structures of most vaccine parts, the information we get from specific methods may have limitations based on data quality and structure details (177, 178). Also, the computer methods we use to study structures have limitations depending on how accurate the models are and the assumptions we make during the analysis (179).

Effective immune responses rely on strong interactions between antigens and immune receptors, such as TLRs (180, 181), with TLR4 being a significant activator of innate immunity. However, other pattern recognition receptors (PRRs), such as RIG-I-like receptors (RLRs) and cytosolic DNA sensors, also play pivotal roles in recognizing HIV and initiating immune responses by triggering signaling pathways that lead to the production of type I interferons and pro-inflammatory cytokines, essential for activating both innate and adaptive immune responses against HIV infection (182, 183). Moreover, dendritic cells (DCs) express chemokine receptors such as CCR5 and CXCR4, which are crucial for HIV entry and influence antigen presentation and T-cell activation (184). Enhancing DC functionality through targeted therapies or vaccines could complement the activation of multiple arms of the immune system. Furthermore, immune checkpoint receptors such as PD-1 and TIM-3 play a role in regulating T-cell responses during chronic HIV infection, suggesting that combining immune checkpoint blockade with vaccine strategies may improve T-cell functionality and overall immune responses (185, 186).

The computational molecular docking and dynamics simulations endorsed stable interactions between the vaccine and these receptors. The optimal vaccine-TLR4 docked complex confirmed the binding affinity of -1181.2 kcal/mol. This complex was characterized by 39 hydrogen bonds and 514 non-bonded contacts. Electrostatic and van der Waals energies play a crucial role, as evidenced by the observed hydrogen bonds and minimal fluctuations, which indicate the vaccine’s potential to induce a significant immune response. The limitations of rigid docking in capturing the dynamic nature of molecular interactions highlight the importance of incorporating flexible docking methods, such as AutoDock and FlexX, which account for both ligand and receptor flexibility. These approaches, combined with molecular dynamics simulations and normal mode analysis, provide deeper insights into conformational changes and binding affinities (187). To gain deeper insights into the complex’s dynamics and stability, the iMODS tool facilitated an analysis of normal modes. The results offered invaluable information regarding the mobility of residues and the rigidity of the vaccine-TLR4 complex. Of particular significance, the eigenvalue of 1.582925 × 10^-07^ underscored the complex’s susceptibility to deformation, with a lower eigenvalue indicating a greater degree of deformability. MDS provides valuable dynamic information; it is essential to complement these findings with experimental validations, such as *in vitro* assays, to assess the actual efficacy of the designed vaccine. Experimental validations can provide crucial insights into the functional characteristics and immunogenicity of the vaccine construct, enhancing the overall assessment of its potential efficacy (188).

Continuing our comprehensive analysis, we optimized the vaccine’s codon sequence for efficient expression in the *E. coli* strain using the JCat tool, achieving a 56.17% GC content. This ensured efficient expression with a CAI (codon adaptation index) value of 1.0. The optimized vaccine sequence was adeptly incorporated into the *E. coli* vector PET28a(+) between the PshAI and HpaI restriction sites, resulting in a construct measuring 5.2 kbp. Lastly, we conducted an in-depth immune simulation analysis using C-ImmSim to evaluate the vaccine’s impact on both humoral and cellular immune responses. The results revealed a substantial stimulation of the primary humoral immune response (189), characterized by increased neutralizing immunoglobulins, including IgG, IgG1, IgG2, and IgM, following each of the three doses. Although immunoglobulin concentrations initially surged post-vaccination, they gradually diminished over time, only to surge markedly again after the third dose. Similarly, various B-cell and T-cell populations exhibited sustained elevation, while the plasma B-cell concentration increased temporarily following vaccination, leading to cellular immunity (190, 191). Combining humoral and cellular immune responses enhances HIV vaccine efficacy by providing stronger protection, reducing the need for high antibody (Ab) levels through vaccine optimization, standardizing Ab measurements, and modulating immune responses (192). It also ensures prolonged immunity by enhancing immune memory, adopting a healthy lifestyle, using immune-boosting supplements, and addressing immune aging (189, 190, 193, 194). Our analysis revealed that T regulatory (Treg) cell activity increases significantly shortly after vaccination (**Figure 10**). This early elevation suggests that the vaccine may promote an immunoregulatory environment, crucial for maintaining immune homeostasis and preventing excessive inflammation or autoimmunity. The heightened Treg response could help balance effector T cell activation, ensuring a robust immune response while minimizing adverse reactions, thereby enhancing long-term immune memory and vaccine safety. These findings underscore the importance of Treg dynamics in developing effective and safe immunization strategies against HIV-1. Dendritic cells, macrophages, and epithelial presenting cells exhibited dynamic fluctuations, and the levels of various cytokines and interleukins increased following vaccination. These findings offer profound insights into the potential efficacy of our vaccine construct in eliciting a robust immune response.

Since our current research is primarily focused on in-silico prediction, questions may arise regarding whether the epitopes presented by supertype MHC molecules can elicit virus-reactive, but not necessarily protective, immunity (195–197). This concern is particularly relevant given the complexities of inducing bnAbs against HIV-1 gp120. While our strategy emphasizes conserved linear epitopes, these may not adequately present neutralizing Abs targets in their proper conformations, a critical factor for effective humoral immunity. The structural characteristics of HIV envelope glycoproteins, including extensive glycosylation and variable loops that obscure bnAb epitopes, complicate vaccine design (198, 199). Notably, only a tiny fraction of antibodies generated in response to viruses are truly viral-neutralizing. Recent advancements, such as the use of stabilized Env trimers, have shown promise in preclinical studies by facilitating the induction of bnAb lineages (200, 201).

Expanding beyond monomeric gp120 and integrating its structural insights with SOSIP trimer strategies hold promise for enhancing bnAb induction. Glycan engineering plays a crucial role in epitope focusing, as modifications such as the addition of N301/N332 to BG505 SOSIP.664 help suppress off-target V3 responses while preserving key neutralizing epitopes, a strategy inspired by hyperglycosylated gp120 mutants like N448Q/E (202, 203). The glycans in the HIV-1 envelope glycoprotein gp120, constituting approximately 50% of its molecular mass, play a critical role in structural stability, functionality, and immune evasion, significantly influencing vaccine design (204, 205). These glycans modulate the flexibility of key antibody recognition loops, such as V1/V2 and V3, while also serving as targets for bnAbs that recognize glycan-dependent epitopes (205, 206). Certain conserved glycans stabilize gp120 and enhance multivalent interactions with bnAbs, making them promising targets for vaccine development (205, 207). Understanding the glycan profiles of gp120 across HIV-1 clades is crucial for designing vaccines capable of eliciting robust, cross-clade immune responses (207). Incorporating glycans into vaccine formulations could improve immunogenicity by closely mimicking the viral structure and addressing immune evasion through glycan shielding.

At the same time, future studies will focus on the detailed mapping of glycan regions on gp120 to enhance our understanding of their impact on epitope accessibility, bnAb interactions, and immune response modulation, thereby advancing HIV vaccine strategies. Similarly, immune complex vaccines leveraging gp120-CD4 interactions, exemplified by IHV01, can stabilize CD4-induced epitopes, and applying this principle to SOSIP trimers (e.g., DS-SOSIP) may enhance bnAb precursors targeting the CD4-binding site, including VRC01-class antibodies (43). Germline-targeting approaches, such as the use of simplified gp120 fragments like eOD-GT8, provide an initial priming step to activate bnAb precursors, which can then be guided through affinity maturation using SOSIP-based booster immunizations, as evidenced in sequential immunization trials (208, 209).

Practical applications of these strategies include glycan occlusion techniques, where masking immunodominant V3 loops in SOSIP trimers reduces non-neutralizing antibody responses (203), and stabilized complexes, where CD4-mimetic modifications in DS-SOSIP trimers improve CD4bs epitope exposure (43). Additionally, heterologous prime-boost regimens employing initial gp120 priming followed by SOSIP trimer boosting have shown enhanced tier 2 neutralization responses in rabbit models (208, 210). These integrated approaches bridge the gap between monomeric gp120 insights and next-generation SOSIP trimer vaccine designs, advancing strategies for bnAb induction.

To assess the actual benefit of this approach for human populations, further *in-vivo* experiments are required to confirm the safety and effectiveness of the proposed vaccine conjugate. However, evaluating the vaccine’s functionality with human HLA-based vaccines is challenging using standard lab animals, including humanized mice (211, 212). Nevertheless, it is possible to conduct *in-vitro* tests to determine if the generated antibodies are neutralizing and to assess the cytolytic potential of T cells (213, 214). Our study emphasizes linear epitopes due to the current challenges in accurately predicting conformational epitopes solely from sequence data. Advances in cryo-electron microscopy and deep learning are expected to facilitate the future integration of structural epitopes into computational vaccine design (215–220). Furthermore, *in vivo* validation using knock-in murine models, such as BG18 or 3BNC117 BCR knock-ins, will be crucial in assessing whether our conserved linear epitopes can effectively complement lineage-targeting immunogens to enhance broadly neutralizing antibody responses (216, 219, 221). Additionally, while prioritizing T-cell responses is vital for controlling HIV infection, a comprehensive vaccine strategy must integrate both T-cell and B-cell responses for optimal protection (222, 223). Future iterations of our vaccine could benefit from incorporating additional HIV antigens such as Gag and Pol, which elicit strong cytotoxic T lymphocyte responses, complementing the immune landscape necessary for effective HIV prevention. This multifaceted approach addresses the limitations of solely targeting gp120 epitopes and may enhance overall vaccine efficacy.

## 5 Conclusion

This bioinformatic-driven research has yielded a promising anti-HIV-1 vaccine candidate, emphasizing the potential of innovative approaches in vaccine development. We selected crucial epitopes from the gp120 protein, designed a structurally sound vaccine, and computationally validated its stability and immunogenicity. Looking ahead, the promising results of our research pave the way for further experimental validation, including preclinical studies and clinical trials. These steps are crucial for assessing the vaccine’s safety and efficacy in real-world scenarios, ultimately bringing us closer to the goal of an effective HIV-1 vaccine. Additionally, the methodologies and insights gained from this study could be applied to developing vaccines against other challenging infectious diseases, showcasing the broader impact of our research in vaccinology.

## Data Availability

The original contributions presented in the study are included in the article/**Supplementary Material**. Further inquiries can be directed to the corresponding authors.
