# Supplementary material for: Immunoinformatic-driven design and evaluation of multi-epitope mRNA vaccine targeting HIV-1 gp120
Source: Front Immunol. 2025 May 13;16:1480025. doi: 10.3389/fimmu.2025.1480025 (PMC12106336; doi:10.3389/fimmu.2025.1480025)
Supplement: Supplementary file 1 [file DataSheet1.docx]

***Supplementary Material***

Immunoinformatic-driven design and evaluation of multi-epitope mRNA vaccine targeting HIV-1 gp120

Muhammad Zeeshan Ahmed^1,2^, Tazeen Rao^1^, Zeeshan Mutahir^2^, Sarfraz Ahmed^3^, Najeeb Ullah^1*^ and Suvash Chandra Ojha^4*^

^1^Department of Biochemistry, Bahauddin Zakariya University, Multan 60800, Pakistan

^2^School of Biochemistry and Biotechnology, University of the Punjab, Lahore 54590, Pakistan

^3^Wellman Center for Photomedicine, Massachusetts General Hospital, Harvard Medical School, Boston, MA 02114, USA

^4^Department of Infectious Diseases, The Affiliated Hospital of Southwest Medical University, Luzhou, Sichuan 646000, China

*** Correspondence:**

Najeeb Ullah

[**najeebkhattak@bzu.edu.pk**](mailto:najeebkhattak@bzu.edu.pk)

Suvash Chandra Ojha

[**suvash_ojha@swmu.edu.cn**](mailto:suvash_ojha@swmu.edu.cn)

**Supplementary Table 1: mRNA vaccine construct sequence for HIV-1 gp120 starts with 5′ m7GCap (green), 5′ UTR (yellow), Kozak sequence (red), Signal peptide (tPA) (mustard), adjuvant (cyan), the protein part of the vaccine consisting of T-cells and B-cells epitopes with linkers, MITD sequence and 6-H tag (black), 3′ UTR (dark green), and Poly (A) tail (purple)**

| **Vaccine** | **Sequence** |
| --- | --- |
| **mRNA** | m7GpppmACAUUUGCUUCUGACACAACUGUGUUCACUAGCAACCUCAAACAGACACCGCCGCCAUGAUGGAUGCGAUGAAACGCGGCCUGUGCUGCGUGCUGCUGCUGUGCGGCGCGGUGUUUGUGAGCCCGAGCCAGGAAAUUCAUGCGCGCUUUCGCCGCGGCGCGCGCAGCUAUCAGGUGAUUUGCCGCGAUGAAAAAACCCAGAUGAUUUAUCAGCAGCAUCAGAGCUGGCUGCGCCCGGUGCUGCGCAGCAACCGCGUGGAAUAUUGCUGGUGCAACAGCGGCCGCGCGCAGUGCCAUAGCGUGCCGGUGAAAAGCUGCAGCGAACCGCGCUGCUUUAACGGCGGCACCUGCCAGCAGGCGCUGUAUUUUAGCGAUUUUGUGUGCCAGUGCCCGGAAGGCUUUGCGGGCAAAUGCUGCGAAAUUGAUACCCGCGCGACCUGCUAUGAAGAUCAGGGCAUUAGCUAUCGCGGCACCUGGAGCACCGCGGAAAGCGGCGCGGAAUGCACCAACUGGAACAGCAGCGCGCUGGCGCAGAAACCGUAUAGCGGCCGCCGCCCGGAUGCGAUUCGCCUGGGCCUGGGCAACCAUAACUAUUGCCGCAACCCGGAUCGCGAUAGCAAACCGUGGUGCUAUGUGUUUAAAGCGGGCAAAUAUAGCAGCGAAUUUUGCAGCACCCCGGCGUGCAGCGAAGGCAACAGCGAUUGCUAUUUUGGCAACGGCAGCGCGUAUCGCGGCACCCAUAGCCUGACCGAAAGCGGCGCGAGCUGCCUGCCGUGGAACAGCAUGAUUCUGAUUGGCAAAGUGUAUACCGCGCAGAACCCGAGCGCGCAGGCGCUGGGCCUGGGCAAACAUAACUAUUGCCGCAACCCGGAUGGCGAUGCGAAACCGUGGUGCCAUGUGCUGAAAAACCGCCGCCUGACCUGGGAAUAUUGCGAUGUGCCGAGCUGCAGCACCUGCGGCCUGCGCCAGUAUAGCCAGCCGCAGUUUCGCAUUAAAGGCGGCCUGUUUGCGGAUAUUGCGAGCCAUCCGUGGCAGGCGGCGAUUUUUGCGAAACAUCGCCGCAGCCCGGGCGAACGCUUUCUGUGCGGCGGCAUUCUGAUUAGCAGCUGCUGGAUUCUGAGCGCGGCGCAUUGCUUUCAGGAACGCUUUCCGCCGCAUCAUCUGACCGUGAUUCUGGGCCGCACCUAUCGCGUGGUGCCGGGCGAAGAAGAACAGAAAUUUGAAGUGGAAAAAUAUAUUGUGCAUAAAGAAUUUGAUGAUGAUACCUAUGAUAACGAUAUUGCGCUGCUGCAGCUGAAAAGCGAUAGCAGCCGCUGCGCGCAGGAAAGCAGCGUGGUGCGCACCGUGUGCCUGCCGCCGGCGGAUCUGCAGCUGCCGGAUUGGACCGAAUGCGAACUGAGCGGCUAUGGCAAACAUGAAGCGCUGAGCCCGUUUUAUAGCGAACGCCUGAAAGAAGCGCAUGUGCGCCUGUAUCCGAGCAGCCGCUGCACCAGCCAGCAUCUGCUGAACCGCACCGUGACCGAUAACAUGCUGUGCGCGGGCGAUACCCGCAGCGGCGGCCCGCAGGCGAACCUGCAUGAUGCGUGCCAGGGCGAUAGCGGCGGCCCGCUGGUGUGCCUGAACGAUGGCCGCAUGACCCUGGUGGGCAUUAUUAGCUGGGGCCUGGGCUGCGGCCAGAAAGAUGUGCCGGGCGUGUAUACCAAAGUGACCAACUAUCUGGAUUGGAUUCGCGAUAACAUGCGCCCGAUGAAAAACGCGCGCACCACCCUGAUUGCGGCGGCGAUUGCGGGCACCCUGGUGACCACCAGCCCGGCGGGCAUUGCGAACGCGGAUGAUGCGGGCCUGGAUCCGAACGCGGCGGCGGGCCCGGAUGCGGUGGGCUUUGAUCCGAACCUGCCGCCGGCGCCGGAUGCGGCGCCGGUGGAUACCCCGCCGGCGCCGGAAGAUGCGGGCUUUGAUCCGAACCUGCCGCCGCCGCUGGCGCCGGAUUUUCUGAGCCCGCCGGCGGAAGAAGCGCCGCCGGUGCCGGUGGCGUAUAGCGUGAACUGGGAUGCGAUUGCGCAGUGCGAAAGCGGCGGCAACUGGAGCAUUAACACCGGCAACGGCUAUUAUGGCGGCCUGCGCUUUACCGCGGGCACCUGGCGCGCGAACGGCGGCAGCGGCAGCGCGGCGAACGCGAGCCGCGAAGAACAGAUUCGCGUGGCGGAAAACGUGCUGCGCAGCCAGGGCAUUCGCGCGUGGCCGGUGUGCGGCCGCCGCGGCGAAGCGGCGGCGAAAAUUGAACCGCUGGGCAUUGCGCCGACCCGCGCGAAACGCCGCGUGGUGGAACGCAAAAAAAAACAGCAGAAAGUGCAUGCGCUGUUUUAUCGCCUGGAUAUUGUGGCGGCGUAUCAGCAGAAAGUGCAUGCGCUGUUUUAUCGCCUGGAUAUUGUGCAGGCGGCGUAUCAGAAAGUGCAUGCGCUGUUUUAUCGCCUGGAUAUUGUGCAGAUUGCGGCGUAUAGCCUGGCGGAAGAAGAAAUUAUUAUUCGCAGCGAAAACCUGACCGCGGCGUAUAUUCGCAGCGAAAACCUGACCAACAACGUGAAAACCAUUAUUGUGGGCCCGGGCCCGGGCCAGCAGAAAGUGCAUGCGCUGUUUUAUGGCCCGGGCCCGGGCAUUACCAUUGGCCCGGGCCAGGUGUUUGGCCCGGGCCCGGGCUGGCAGGGCGUGGGCCAGGCGAUGUAUGGCCCGGGCCCGGGCGCGCCGACCCGCGCGAAACGCCGCGUGGGCCCGGGCCCGGGCAUUGUGGGCAUUGUGGCGGGCCUGGCGGUGCUGGCGGUGGUGGUGAUUGGCGCGGUGGUGGCGACCGUGAUGUGCCGCCGCAAAAGCAGCGGCGGCAAAGGCGGCAGCUAUAGCCAGGCGGCGAGCAGCGAUAGCGCGCAGGGCAGCGAUGUGAGCCUGACCGCGCAUCAUCAUCAUCAUCAUGCUCGCUUUCUUGCUGUCCAAUUUCUAUUAAAGGUUCCUUUGUUCCGUAAGUCCAACUACUAAACUGGGGGAUAUUAUGAAGGGCCUUGAGCAUCUGGAUUCUGCCUAAUAAAAAACAUUUAUUUUCAUUGCGCUCGCUUUCUUGCUGUCCAAUUUCUAUUAAAGGUUCCUUUGUUCCGUAAGUCCAACUACUAAACUGGGGGAUAUUAUGAAGGGCCUUGAGCAUCUGGAUUCUGCCUAAUAAAAAACAUUUAUUUUCAUUGCAAAAAAAAAAAAAAAAAAAAAAAAAAAAAAAAAAAAAAAAAAAAAAAAAAAAAAAAAAAAAAAAAAAAAAAAAAAAAAAAAAAAAAAAAAAAAAAAAAAAAAAAAAAAAAAAAAAAAAAA |

**Supplementary Table 2. Physicochemical properties, antigenicity, allergenicity, toxicity, and solubility of vaccine construct.**

| **Physiochemical properties** | **Vaccine construct** | **Servers utilized** |
| --- | --- | --- |
| **Molecular weight (kDa)** | 42.2 | ExPASy-ProtParam |
| **Number of amino acids** | 406 |  |
| **Theoretical pI** | 9.14 |  |
| **Formula** | C_1882_H_2959_N_547_O_553_S_6_ |  |
| **Ec (M^-1^ cm^-1^, at 280nm)** | 46995 |  |
| **Estimated half-life** | >10 hours (*E. coli*, *in vivo*)  >20 hours (yeast, *in vivo*)  30 hours (mammalian reticulocytes, *in vitro*) |  |
| **Antigenicity Score** | 0.6316, 0.93 | VaxiJen 2.0, ANTIGENpro |
| **Antigenicity** | Antigenic |  |
| **Allergenicity** | Non-Allergenic | AllerTop 2.0 |
| **Toxicity** | Non-Toxic | Toxinpred2 |
| **Solubility score** | 0.697 | SoluProt |

**Supplementary Table 3. Docking and MDS Parameters of the Vaccine-TLR4 Complex, highlighting key interactions and stability metrics throughout the simulation.**

| **Parameter** | **Value/Range** | **Observation** |
| --- | --- | --- |
| **Docking Parameters** | | |
| **Number of Interface Residues** | 46 (TLR4) – 36 (Vaccine) | Indicates strong interaction at the interface. |
| **Interface Area (Å²)** | 1847–1993 | Significant interface area, contributing to stable binding. |
| **Binding Affinity (kcal/mol)** | -1181.2 | Represents a highly favorable binding energy. |
| **Electrostatic-favored Binding Affinity (kcal/mol)** | -1270.3 | Electrostatic interactions significantly contribute to binding. |
| **Hydrophobic-favored Binding Affinity (kcal/mol)** | -1505.9 | Hydrophobic interactions enhance binding stability. |
| **Van-der Waals and Electrostatic Binding Affinity (kcal/mol)** | -322.7 | Reflects combined contributions of van-der Waals and electrostatic forces. |
| **Number of Salt Bridges** | 2 | Indicates specific electrostatic interactions at the interface. |
| **Number of Hydrogen Bonds** | 20 | Key factor in stabilizing the docked complex. |
| **Number of Non-bonded Contacts** | 223 | Indicates extensive interaction network between vaccine and TLR4. |
| **MDS Parameters** | | |
| **Simulation Duration** | 100 ns | Analyzed vaccine-TLR4 interactions over the entire simulation time. |
| **RMSD (C-alpha atoms, vaccine-TLR4 complex)** | 17.5–19 Å | Stabilized after 40 ns and remained stable throughout. |
| **RMSD (TLR4 receptor)** | 1.5–3.5 Å | Showed stability from the start of the simulation. |
| **RMSD (Vaccine)** | 25–29 Å | Stabilized after 40 ns, following the same pattern as the vaccine-TLR4 complex. |
| **RMSF (Vaccine-TLR4 complex)** | Peaks at residue index ~600 | Higher flexibility observed in the C-terminal zone of the vaccine, indicating potential interactions. |
| **RMSF (TLR4 receptor)** | Low fluctuation | Indicates high stability of TLR4 in the docked complex. |
| **Number of Hydrogen Bonds (MDS)** | Increased over time | Confirms the stability of the docked vaccine-TLR4 complex. |
| **Secondary Structure Elements (SSE)** | 22.04% | Comprised 8.7% helix (red peaks) and 13.34% strand (blue peaks). |


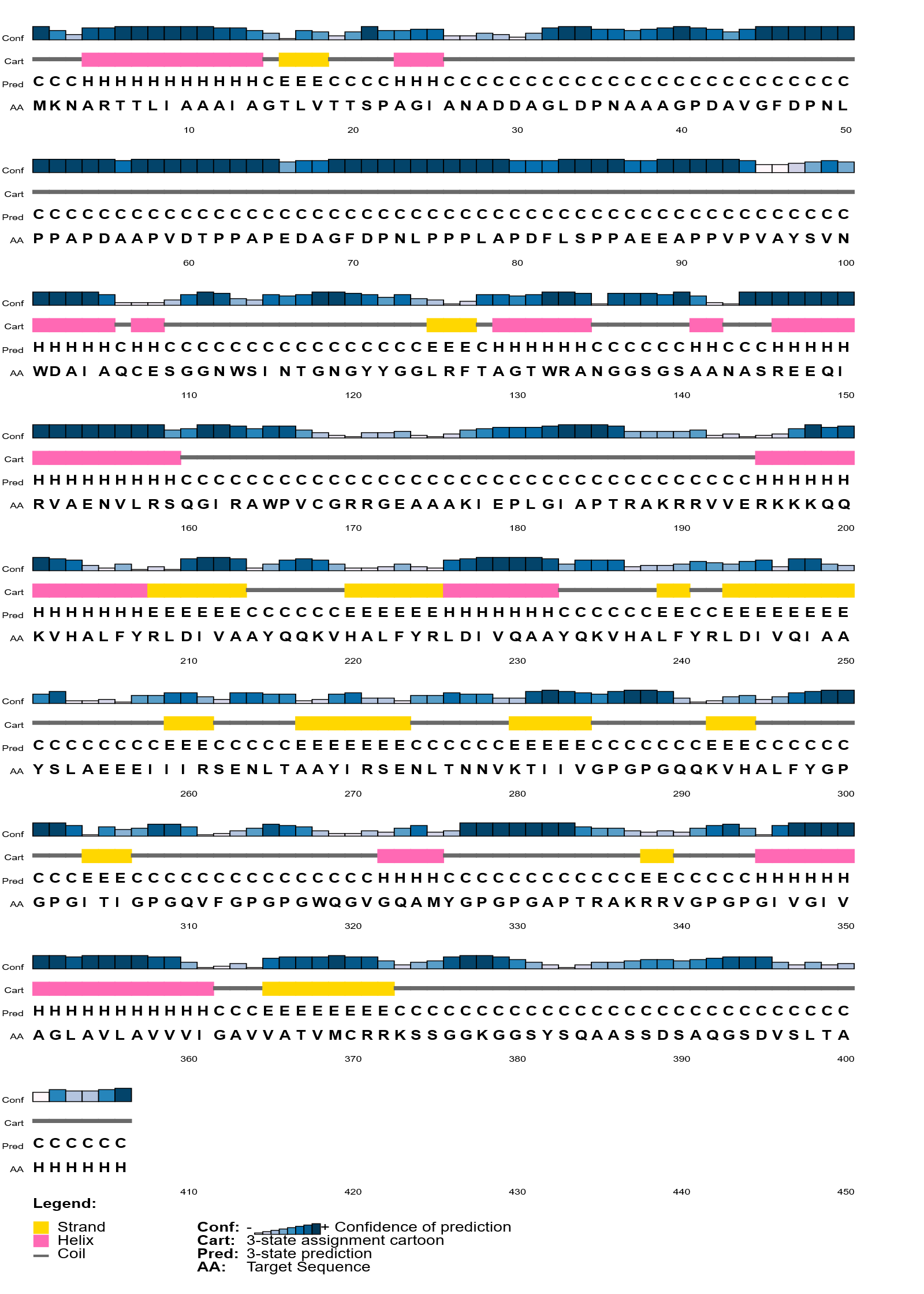


**Supplementary Figure 1. The secondary structure of** **the translated HIV-1 gp120 vaccine is shown in PSIPRED.**


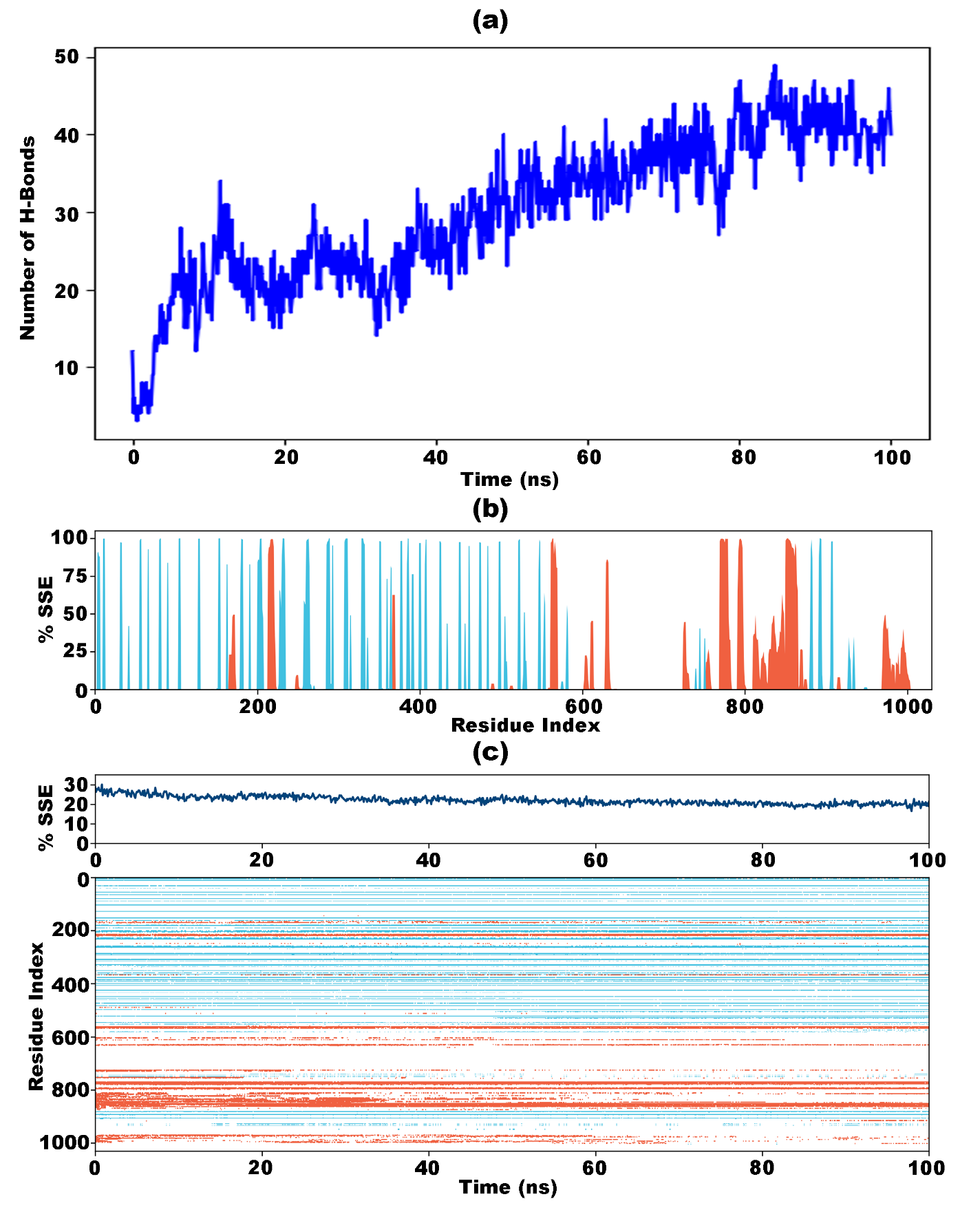


**Supplementary Figure 2. (a) The number of hydrogen bonds between the vaccine and TLR4 docked complex increased over the simulation time, indicating enhanced complex stability. (b-c) The docked complex's secondary structure elements (SSE) show that 22.04% comprises secondary features, including 8.7% helix and 13.34% strand, with variations across time intervals and residue indices.**
